# Hypomagnesemia: A Clinical and Nutritional Update

**DOI:** 10.1007/s13668-026-00745-5

**Published:** 2026-03-24

**Authors:** Anastasia Papagiannidou, Maria Mitropoulou, Konstantinos Papantzikos, Dimitra Petropoulou, Dimitrios Tsilingiris, Faidon Magkos, Maria Dalamaga

**Affiliations:** 1 Department of Biological Chemistry, Medical School, National and Kapodistrian University of Athens, Athens, 11527 Greece; 2 First Department of Internal Medicine, University Hospital of Alexandroupolis, Democritus University of Thrace, Alexandroupolis, Greece; 3 Department of Nutrition, Exercise and Sports, University of Copenhagen, Rolighedsvej 26, Frederiksberg C, 1958 Denmark

**Keywords:** Cardiovascular disease, Gut microbiome, Hypomagnesemia, Magnesium, Magnesium deficiency, Micronutrient deficiency, Nutrition, Supplementation, TRPM6/7

## Abstract

**Purpose of Review:**

Hypomagnesemia, defined as low serum/plasma magnesium concentration, is a highly prevalent yet underrecognized electrolyte disorder with extensive clinical, metabolic, and nutritional implications. This review provides an updated synthesis of magnesium physiology, dietary determinants, homeostatic regulation, diagnostic challenges, and therapeutic strategies, with particular emphasis on recent meta-analyses and large-scale epidemiological evidence linking hypomagnesemia to multisystem disease.

**Recent Findings:**

Accumulating evidence has shown consistent associations between low serum or dietary magnesium and increased risk of cardiometabolic disorders (hypertension, type 2 diabetes mellitus, metabolic syndrome, and cardiovascular disease), neuropsychiatric conditions (migraine, depression, cognitive impairment, and dementia), osteoporosis, immune dysregulation, and adverse outcomes in hospitalized, critically ill, and chronic kidney disease patients. Mechanistic studies have clarified the roles of TRPM6/7 channels, tight junction claudins, and basolateral magnesium transporters in intestinal and renal magnesium handling, elucidating pathways underlying both inherited and acquired deficiencies. Research has also highlighted the contribution of modern dietary patterns, food processing, mineral-depleted drinking water, medication use (notably proton pump inhibitors, diuretics and chemotherapeutic agents), and gut microbiome alterations to widespread subclinical deficiency. Meta-analyses of RCTs indicate that magnesium supplementation confers modest but clinically relevant improvements in blood pressure, glycemic control, inflammatory markers, endothelial function, migraine frequency, and depressive symptoms, particularly in individuals with baseline hypomagnesemia. However, serum magnesium remains an insensitive biomarker of total body magnesium status, and consensus on optimal diagnostic thresholds and replacement strategies is lacking.

**Summary:**

Magnesium deficiency contributes to a wide spectrum of multisystem disorders, and is driven by dietary insufficiency, gastrointestinal and renal losses, medication use, chronic disease, and altered microbiome function. Meta-analytic evidence supports its role as a modifiable risk factor across cardiovascular, metabolic, neurological, skeletal, and immune disorders. Dietary modification, optimized supplementation, and correction of underlying causes of deficiency remain central to management. Future research should focus on improved diagnostic tools, personalized dosing approaches and long-term outcomes of magnesium repletion. Enhancing clinical awareness and integrating magnesium evaluation into routine care may reduce the growing burden of hypomagnesemia.

## Introduction

Magnesium (Mg or Mg^2+^, Mg^++^), described previously as the “forgotten electrolyte”, is a fundamental element for living organisms being the fourth most abundant mineral in the human body, following sodium (Na or Na^+^), potassium (K or K^+^) and calcium (Ca or Ca^2+^, Ca^++^), and the second most abundant cation in the intracellular fluid after potassium [1].

The overall content of magnesium in the human body is estimated to be around 20 mmol per kg of fat-free tissue. For an average 70 kg adult with approximately 20% body fat, this corresponds to a total magnesium level ranging from about 1,000 to 1,120 mmol, or roughly 25 g [2]. Of this, 99% is distributed intracellularly in the bone (60%), skeletal muscle (29%), and non-muscular soft tissues such as heart and liver (10%) [3]. In the intracellular space, magnesium is mainly bound to proteins and negatively charged molecules such as nucleic acids and adenosine triphosphate (ATP), while only 1–5% of the intracellular magnesium exists as free magnesium [2]. The remaining 1% of magnesium is present extracellularly, either ionized (50%), protein-bound (30%), or complexed with anions such as phosphate, bicarbonate, sulfate, and citrate (20%) [4]. Only the ionized form of magnesium is considered biologically active. Intracellular magnesium levels typically range between 5 and 20 mmol/L, while serum magnesium concentration is approximately 0.6–1.1 mmol/L [2, 5]. Maintaining adequate magnesium is essential due to its wide-ranging structural and physiological roles.

Magnesium is a critical element in numerous cellular and physiological processes, primarily due to its nucleotide-binding capacity and regulatory influence in enzymatic activity. All ATPase-dependent reactions necessitate the formation of Mg^2+^-ATP complexes, including those essential for nucleic acid synthesis and function. As a ubiquitous enzymatic cofactor, magnesium supports the activity of hundreds of enzymes across all cell types. In addition, magnesium is essential for the regulation of glucose, lipid and protein metabolism. It contributes significantly to neuromuscular excitability, cardiac electrophysiological stability, vascular tone modulation, synaptic transmission and neuroplasticity, and endocrine signaling [6]. Finally, magnesium functions as a secondary messenger in the intracellular signaling cascades and plays a regulatory role in the expression of circadian-clock genes, thereby contributing to the maintenance of circadian rhythm in biological systems [7].

Although magnesium is vital for optimal cell function, its deficiency is often overlooked by physicians compared to other electrolyte abnormalities [8]. Because most magnesium is intracellular, plasma levels are not routinely assessed in clinical practice. Other important reasons behind poor attention on this ion include the inadequate comprehension of its physiology and the clinical presentation of hypomagnesemia appearing only when magnesium levels are significantly depleted [3]. On the contrary, hypermagnesemia is an uncommon condition that typically arises in individuals with impaired kidney function, who are taking magnesium-retaining drugs. Despite being frequently overlooked, hypomagnesemia may present with severe and potentially life-threatening manifestations, including neuromuscular irritability, musculoskeletal symptoms, and electrocardiographic abnormalities [9]. In many instances, it occurs concurrently with other electrolyte imbalances, such as hypocalcemia and hypokalemia, further complicating the patient’s clinical condition [6, 10].

Despite abundant research on magnesium physiology, current literature lacks an integrated, clinically oriented synthesis linking molecular mechanisms with modern dietary patterns and real-world causes of hypomagnesemia. Recent advances in magnesium transporters, drug-induced renal wasting, and gut microbiome interactions remain scattered across subspecialties. A concise and updated approach to guide clinicians in recognizing, evaluating, and correcting hypomagnesemia is therefore needed. Thus, this review aims to integrate advances in magnesium physiology with contemporary clinical and nutritional evidence, providing a consolidated strategy for understanding the causes, manifestations, diagnosis, and management of hypomagnesemia. By synthesizing mechanistic insights, epidemiological trends, dietary determinants, microbiome interactions, and findings from recent clinical trials, we aim to offer clinicians and nutrition professionals an up-to-date, practical resource for recognizing, preventing, and treating magnesium deficiency. Finally, we also cover practical laboratory assessment and evidence-informed repletion strategies.

## Literature search

A targeted narrative literature search was conducted using PubMed, Scopus, and Google Scholar for studies published up to November 30, 2025. Search terms included “magnesium,” “hypomagnesemia,” “magnesium deficiency,” “magnesium homeostasis,” “TRPM6/7,” “magnesium absorption,” “drug-induced hypomagnesemia,” “dietary magnesium,” “magnesium supplementation,” and “magnesium-related disorders.” Priority was given to recent clinical trials, meta-analyses, large epidemiological studies, and mechanistic papers relevant to nutritional and clinical practice. Additional references were identified through manual screening of bibliographies of key articles and guidelines. Since this is a narrative review, no formal methodological quality assessment or systematic inclusion criteria were applied. Finally, we recognize that the literature on magnesium physiology and hypomagnesemia is extensive, and although we aimed to incorporate the most relevant and high-quality evidence, not all available studies could be discussed in detail within this review.

## The role of magnesium in human physiology

###  Magnesium and intracellular enzymes

Magnesium is a universal element found intracellularly in all living organisms, i.e. from plants to complex animals, being a necessary cofactor for ATP, the primary energy currency of cells. This mineral plays a central part in numerous cellular and physiological functions, largely due to its ability to bind to nucleotides and influence enzyme activity. Magnesium acts as a cofactor for over 600 enzymes and as an activator for 200 enzymes catalyzing diverse reactions [11]. In the cytoplasm, magnesium is required for the pathway of glycolysis playing an important role in the function of hexokinase, phosphofructokinase, phosphoglycerate kinase, pyruvate kinase, aldolase, and enolase [12]. In the mitochondria, the activity of three significant dehydrogenases, such as pyruvate dehydrogenase, and isocitrate dehydrogenase and α-ketoglutarate dehydrogenase in the Krebs cycle, as well as the activation of Fo/F1-ATPase for ATP-synthesis relies on magnesium [13, 14]. Regarding specific tissues, magnesium affects the behavior of the enzyme creatine kinase, which is responsible for phosphocreatine turnover supporting ATP production when the heart and muscle are subjected to a heavy workload [12]. In the liver, magnesium regulates glucose-6 phosphatase and phosphoenolpyruvate carboxykinase, two enzymes involved in gluconeogenesis [15]. Several other protein kinases depend on magnesium, including the tyrosine kinase of β-subunit of the insulin receptor [16].

### Magnesium in nucleic acid metabolism and epigenetic regulation

Magnesium is important to maintain genomic and genetic stability, playing an important role in DNA replication as a cofactor for nuclear enzymes including DNA polymerases, topoisomerases, helicases, exonucleases, and enzymes involved in DNA repair mechanisms, such as DNA polymerase beta, DNA ligases, and DNA endonucleases [17]. Magnesium also stabilizes the natural DNA conformation by decreasing the concentration of negative charges along the DNA molecule [18]. Notably, magnesium contributes to RNA transcription, ribosomal activity, and protein formation playing an important role in cell proliferation, RNA structure formation, and catalytic activity [19, 20]. Finally, magnesium influences epigenetic regulation of gene expression through its effects on chromatin structure, transcriptome modulation, and cellular differentiation [21]. Adequate magnesium intake may beneficially modulate inflammation and oxidative stress through epigenetic mechanisms, including effects on the gut microbiota and systemic health [22]. In particular, magnesium supplementation for 4 weeks in overweight individuals has been shown to alter gene expression profiles, including up- and down-regulation of genes related to metabolic and inflammatory pathways, further supporting its role in epigenetic modulation [23].

### Magnesium and bone formation/metabolism

Serum magnesium concentrations are closely linked to bone formation and metabolism, as there is a constant exchange between bone surface magnesium and blood magnesium [24]. Magnesium plays a regulatory role in determining crystal size and development both by increasing the solubility of phosphate and calcium ions, which are essential for the formation of hydroxyapatite [25], and by inducing the proliferation of osteoblasts [26]. Apart from its direct influence in hydroxyapatite crystals, magnesium contributes to bone health through several other mechanisms involving vitamin D and parathyroid hormone (PTH), linked to calcium concentrations. The activity of hepatic 25-hydroxylase and renal 1-hydroxylase, which are important enzymes for the synthesis of vitamin D, requires magnesium, which is also involved in the inactivation of vitamin D, by stimulating renal 24-hydroxylase [27]. Additionally, magnesium affects PTH secretion in relation to body calcium levels and regulates the sensitivity of the target organs to the PTH signal, which in turn influences the serum concentration of vitamin D [28, 29].

### Magnesium and cardiovascular system

Magnesium plays a critical role in maintaining cardiovascular integrity by regulating blood pressure, stabilizing cardiac rhythm, suppressing inflammation, and preventing thrombosis. These effects are mediated through its involvement in calcium balance, modulation of cardiac muscle activity, and facilitation of endothelial-dependent vascular relaxation. Magnesium regulates intracellular calcium by blocking calcium channels, thus reducing the risk of calcium-induced cytotoxicity [30]. More specifically, magnesium controls the activity of Ca^2+^–ATPases in proportion to calcium and functions as a calcium antagonist by competing for binding sites in proteins and Ca^2+^ transporters [31]. Other membrane ion transporters affected by magnesium include the Na^+^/K^+^–ATPase, K^+^ channels, and the Na^+^–Ca^2+^ exchanger [32]. In blood vessels, magnesium exerts a vasodilatory effect, since it down-regulates calcium channels and up-regulates the endothelial nitric oxide (NO) synthase, thus promoting the production of NO [33]. In addition, magnesium affects the synthesis of the platelet inhibiting factor prostacyclin (PGI_2_) and prostaglandin E1 (PGE_1_), which are also vasodilators [32]. Finally, magnesium may improve lipid profile and endothelial function, as well as decrease free oxygen radicals, by reducing the inflammatory response and oxidative stress, potentially through the inhibition of the interleukin-6 (IL-6) pathway, nuclear factor kappa B (NF-κB) pathway, and L-type calcium channels [34].

### Magnesium and neurological function

Magnesium is essential for proper neurological function, exerting significant influence in neuronal excitability, synaptic transmission, and overall neurophysiological stability. Magnesium enhances the release of NO in the brain, promoting vasodilation and facilitating the regulation of gene transcription and the secretion of neurotransmitters [35]. Moreover, the modulation of the inhibitory gamma-aminobutyric acid (GABA) receptor function and the release of neuropeptides, such as calcitonin gene–related peptide (CGRP) and substance P, is affected by magnesium [36]. It is worth noting that magnesium occupies the calcium channel of the N-methyl-D-aspartate (NMDA) receptor and blocks ion flow at resting membrane potential, thus suppressing the excitatory signals and neuronal plasticity involved in learning and memory [37].

## Nutritional aspects for magnesium

Magnesium is obtained exclusively through the diet, and habitual intake is a major determinant of magnesium status. The average daily magnesium intake generally ranges between 300 and 360 mg per day, often lower than recommended values due to modern dietary habits [38]. According to the U.S. Food and Nutrition Board, the recommended daily allowance (RDA) is 420 mg for men and 320 mg for women, while the European Food Safety Authority (EFSA) has established adequate intake (AI) levels of 350 mg/day for men and 300 mg/day for women [38, 39].

### Dietary sources of magnesium

**Fig. 1:**
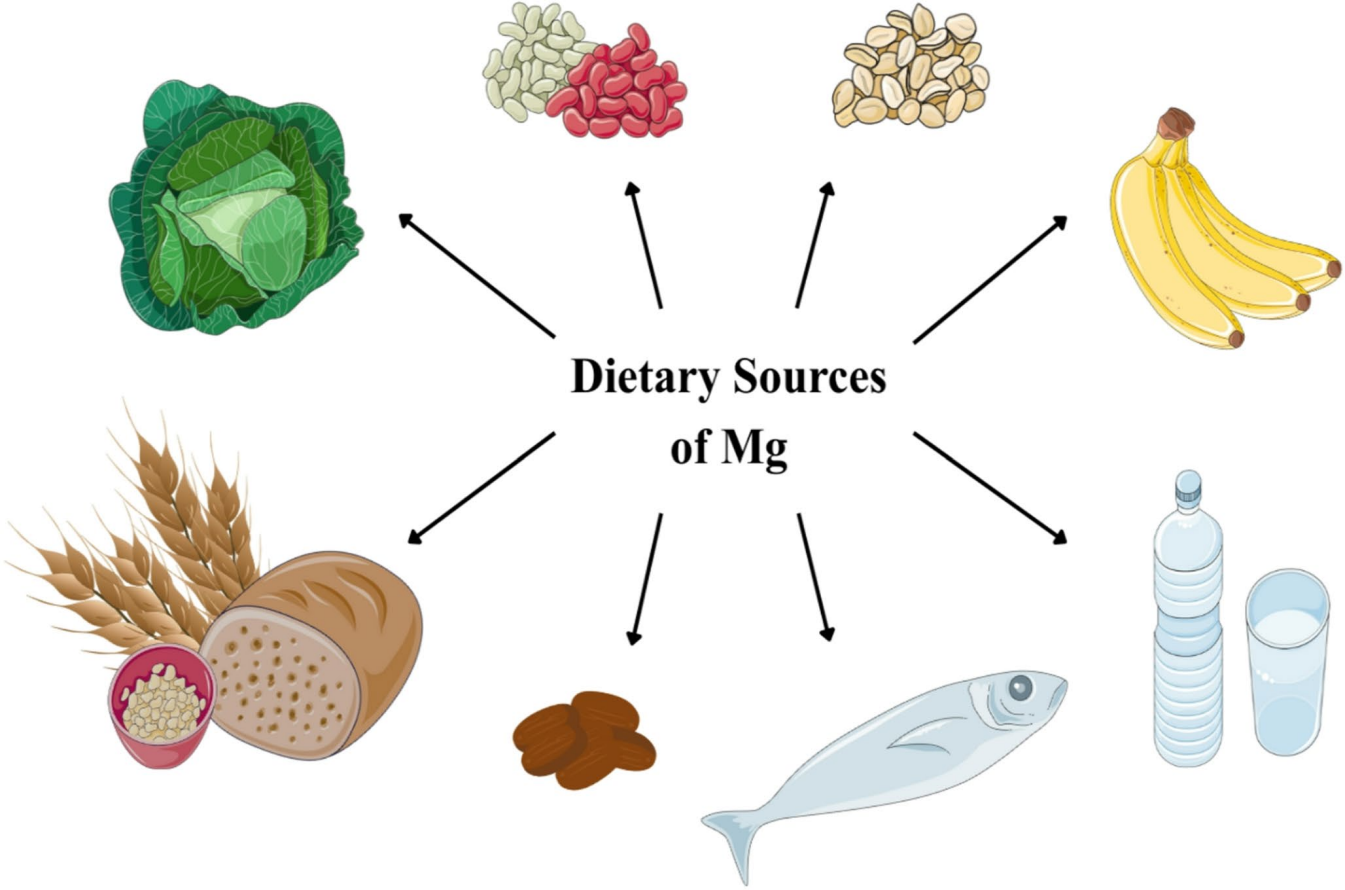
Dietary sources of magnesium include leafy vegetables, seeds, nuts, beans, some fruits, seafood, mineral water and whole-grain products. Parts of the figure are from the free medical site http://smart.servier.com/ by Servier licensed under a Creative Commons BY 4.0 License https://creativecommons.org/licenses/by/4.0/ (accessed on 12 December 2025)

Foods naturally rich in magnesium include green leafy vegetables, where magnesium binds to chlorophyll [25], legumes, beans, nuts (almonds, cashews, Brazil nuts), seeds (pumpkin, cocoa, chia), whole grains, some fruits, and seafood [25, 40] **(**Fig. 1**)**. Some breakfast cereals and fortified foods also contribute meaningfully to dietary magnesium intake [38].

Water may also serve as a source of magnesium, depending on its mineral content. Hard water, which is rich in magnesium and calcium, may deliver approximately 10–30 mg/L of magnesium, whereas soft water contains significantly less. The magnesium content in tap and bottled waters varies widely by geographic region and brand, with reported ranges from < 0.01 up to 128 mg/L in bottled waters and up to 315 mg/L in tap waters, though commonly observed concentrations are towards the lower end of this range [41]. Moreover, filtration processes, especially those involving reverse osmosis, deionization or softening, substantially reduce the mineral content of tap water, including magnesium, often to negligible levels [42].

Some commercially available products deliver meaningful amounts of magnesium and can help maintain AI, particularly in populations with low dietary consumption. In European and North American studies, some bottled waters can provide up to 41% of the adult magnesium dietary reference intake per liter, although most bottled waters contribute much less [43, 44]. The clinical relevance of magnesium intake from water is supported by evidence that higher magnesium concentrations in drinking water are associated with improved serum magnesium levels and may have beneficial effects on cardiovascular health, particularly prevention of stroke in postmenopausal women, and bone health [42, 45]. However, the contribution to total intake is highly dependent on both the mineral content of the water and the volume consumed.

### Effects of food processing and cooking

Modern food processing and cooking methods have a substantial impact on the magnesium content of foods. Refining of grains, such as milling and polishing, removes the mineral-rich outer layers, resulting in a loss of up to 70–80% of magnesium content. Hence, whole grains contain far higher magnesium levels than refined cereals [25]. Industrial processing steps, including soaking and blanching, further decrease magnesium levels due to leaching into processing water [46]. Cooking techniques that use large amounts of water, such as boiling, may cause significant magnesium loss from vegetables, whereas methods like steaming or microwave cooking better preserve mineral content by minimizing direct contact with water and reducing leaching [47]. Overall, the cumulative effect of these practices contributes to the lower magnesium intake observed in populations consuming highly processed foods [48].

### Magnesium intake patterns in modern diets

Population-based studies have consistently shown that a substantial proportion of adults in the USA consume less than the estimated average requirement (EAR) for magnesium, with recent National Health and Nutrition Examination Survey (NHANES) analyses indicating that only about half of adults meet recommended intake levels from diet and supplements combined [49–51]. This shortfall is primarily attributed to Western dietary patterns characterized by the increased consumption of processed foods and refined grains, and the low intake of vegetables, nuts, seeds, and whole grains [49, 50]. In contrast, individuals adhering to plant-forward dietary patterns such as the Mediterranean diet, Dietary Approaches to Stop Hypertension (DASH) diet, and others, have significantly higher magnesium intake due to the emphasis on magnesium-rich foods including legumes, nuts, seeds, and leafy vegetables [52].

Epidemiological data from the NHANES and other large cohorts indicate that higher dietary magnesium intake is associated with a lower prevalence of hypertension, diabetes mellitus (DM), hyperlipidemia, metabolic syndrome, and cardiovascular mortality [52, 53]. For example, US adults in the highest quintile of magnesium intake have markedly reduced odds of hypertension and DM compared to those in the lowest quintile [52]. These findings likely reflect the mechanistic role of magnesium in cardiometabolic health and highlight the importance of dietary patterns that prioritize minimally processed, plant-based foods for optimal magnesium status.

### Gut microbiome and magnesium

The gut microbiome plays a critical role in regulating magnesium homeostasis. Low dietary magnesium intake and proton pump inhibitor (PPI) therapy are associated with reduced gut microbial diversity and altered composition, which may predispose to hypomagnesemia [54]. In murine models, omeprazole treatment under magnesium-deficient conditions leads to decreased serum magnesium and a shift in microbial populations, notably increasing the abundance of *Akkermansia muciniphila* and *Turicibacter sanguinis* [55, 56]. Similarly, magnesium restriction in mice and rats increases alpha diversity and enriches taxa such as *Romboutsia ilealis*, while reducing *Oscillospiraceae* and *Lachnospiraceae* [56].

Microbial fermentation of dietary fibers produces short-chain fatty acids (SCFAs), including butyrate, which directly modulate magnesium transport. Butyrate has been shown to inhibit active magnesium uptake in human colonocytes, independent of metabolic regulation [57]. Conversely, magnesium supplementation in colitic mice increases beneficial bacteria such as *Bifidobacterium* and reduces pro-inflammatory *Enterobacteriaceae*, supporting mucosal health and metabolic homeostasis [58].

Collectively, these findings underscore the bidirectional relationship between magnesium status and the gut microbiome. Dietary magnesium shapes microbial communities, while microbial metabolites such as SCFAs regulate epithelial magnesium transport. Specific taxa, including *Akkermansia*,* Turicibacter*,* Romboutsia*,* Lactobacillus*,* Oscillospiraceae*,* and Lachnospiraceae*, are implicated in these processes, with clinical relevance for conditions such as PPI-induced hypomagnesemia and inflammatory bowel disease [54]. These findings also suggest the potential future role for microbiome-targeted therapies, including prebiotics, probiotics, and novel “next-generation probiotics,” to enhance mineral uptake.

### Dietary strategies to improve magnesium intake

Increasing magnesium intake is best achieved by regularly consuming green leafy vegetables, legumes, whole grains, nuts, and seeds, and by favoring dietary patterns such as the DASH and Mediterranean diets, which are associated with higher magnesium intake and improved blood pressure control [59]. The bioavailability of magnesium is influenced by both food matrix and meal composition. Magnesium absorption is enhanced when consumed with meals and may be further supported by fermentable fibers, such as inulin, which modulate gut microbiota and increase its bioavailability [60, 61]. In addition, magnesium-rich mineral water may be considered in regions with low-magnesium tap water. When dietary intake remains insufficient, oral supplementation may be appropriate, with organic salts preferred for better absorption [6].

## Homeostasis and regulation of magnesium

**Fig. 2:**
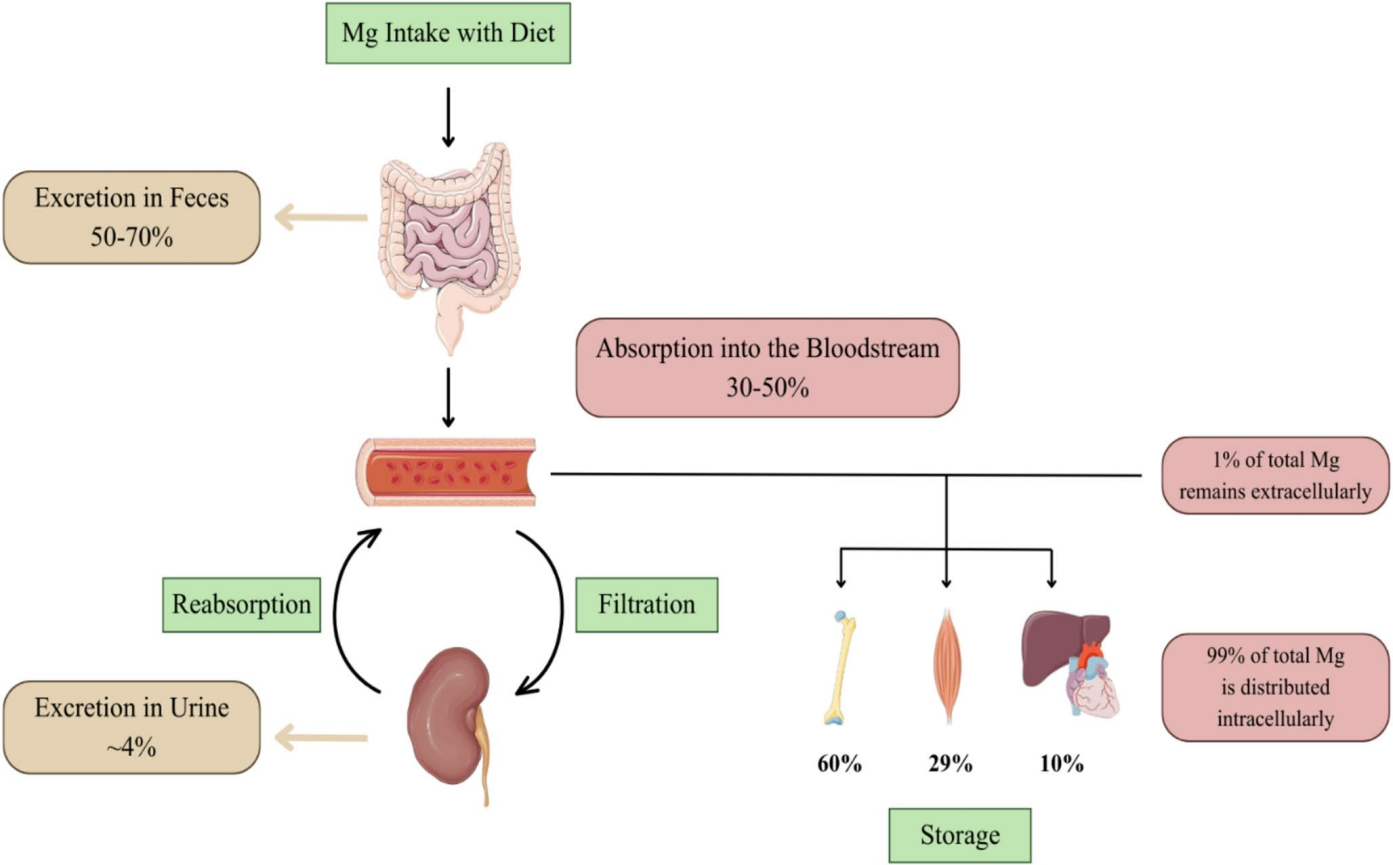
Magnesium homeostasis and distribution. Parts of the figure are from the free medical site http://smart.servier.com/ by Servier licensed under a Creative Commons BY 4.0 License https://creativecommons.org/licenses/by/4.0/ (accessed on 12 December 2025)

 Magnesium homeostasis is governed by a dynamic interplay between intestinal absorption, renal reabsorption, and skeletal storage (Fig.2).

### Intestinal absorption of magnesium

**Fig. 3:**
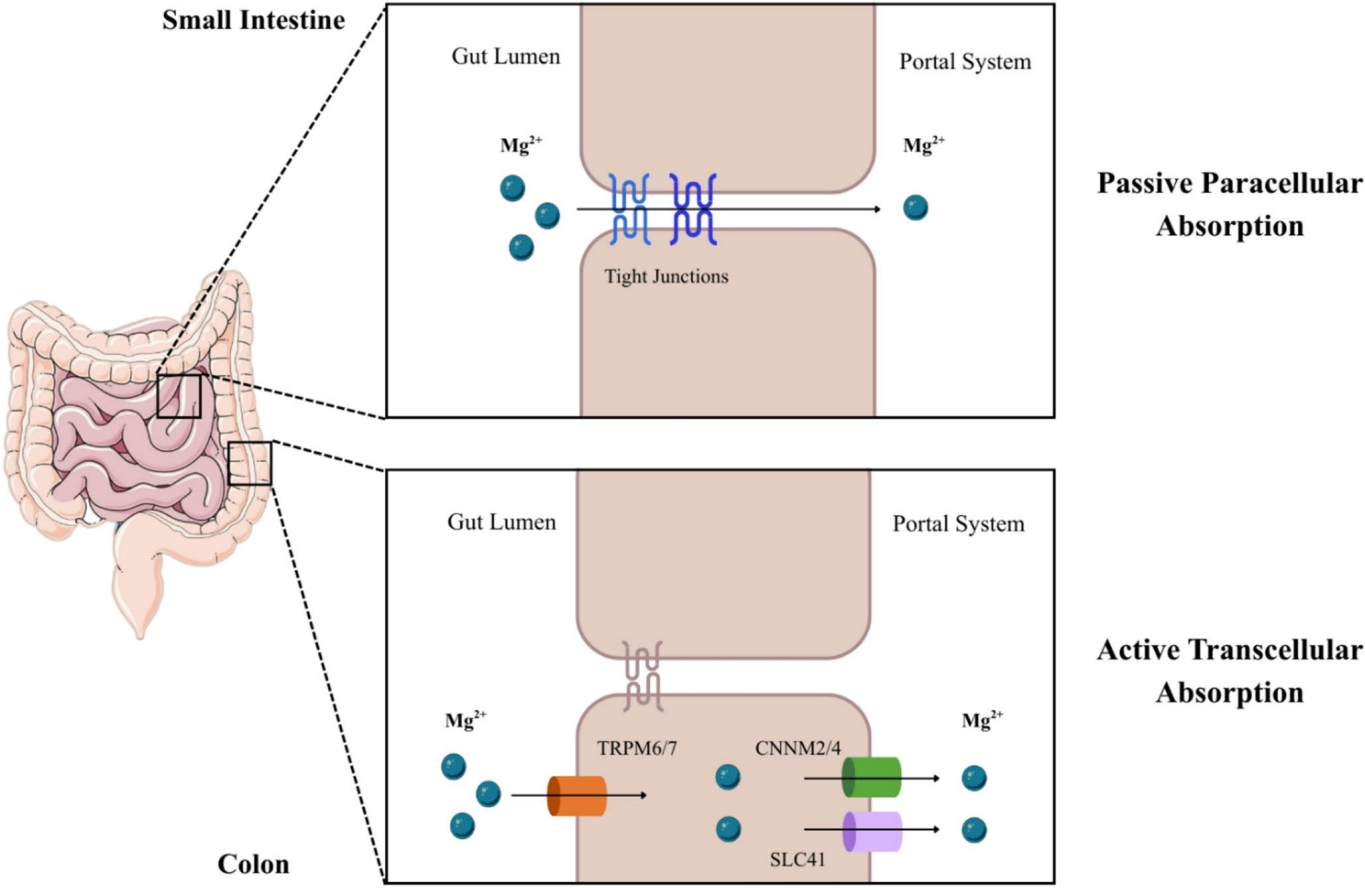
Intestinal Mg absorption is conducted via a passive paracellular mechanism in the small intestine and via an active transcellular mechanism in the colon. Abbreviations: CNNM2/4: cyclin M magnesium transporter 2 and 4; Mg2+: magnesium ion; SLC41: solute carrier family 41; TRPM6/7: transient receptor potential melastatin channels 6 and 7. Parts of the figure are from the free medical site http://smart.servier.com/ by Servier licensed under a Creative Commons BY 4.0 License https://creativecommons.org/licenses/by/4.0/ (accessed on 12 December 2025)

Approximately 30–50% of dietary magnesium is absorbed throughout the gastrointestinal (GI) tract, via two complementary pathways: a saturable active transcellular route mediated by specific transporters, and a non-saturable paracellular route driven by luminal concentration gradients [62]. The small intestine is the principal site of uptake [62], with passive paracellular transport predominating in the jejunum and ileum, while active, transcellular absorption through ion channels of the superfamily transient receptor potential melastatin channels 6 and 7 (TRPM6/7) is more prominent in the distal ileum and colon [63]. Paracellular absorption occurs through tight junctions composed of occludins, claudins, and E-cadherin, which preserve epithelial barrier integrity and regulate ionic permeability [64]. Claudin isoforms (claudin-2, −7, and − 12) enhance cation selectivity and permeability in the duodenum and ileum [65, 66], while the reduced expression of tightening claudins facilitates greater magnesium flux [67]. A lumen-negative transepithelial potential (approximately − 5 mV) further augments passive uptake [66]. TRPM6, localized to the apical membranes of epithelial cells, serves as a key entry channel for magnesium [68], while TRPM7 cooperates in maintaining systemic balance [69, 70]. Efflux pathways from intestinal cells are less well defined, but evidence implicates members of the Solute Carrier Family 41 (SLC41) and cyclin M magnesium transporter (CNNM2, CNNM4) families in basolateral magnesium extrusion [62] **(**Fig. 3**)**. Intestinal absorption is further influenced by mucosal integrity, luminal pH, gut microbiota, and hormones including PTH, estrogen, insulin, epidermal growth factor (EGF), fibroblast growth factor 23 (FGF23), and vitamin D [71]. Notably, 1,25-dihydroxyvitamin D enhances GI uptake [64], while PTH stimulates intestinal absorption, renal reabsorption, and skeletal mobilization, jointly sustaining circulating magnesium levels [72]. Magnesium absorption is also enhanced by low dietary magnesium intake; increased intake of prebiotic fibers (e.g. inulin), which undergo fermentation to SCFAs that acidify the lumen and increase magnesium solubility; protein ingestion; and the presence of certain pharmacologic agents such as sodium–glucose cotransporter 2 (SGLT2) inhibitors [73]. Conversely, absorption is reduced by high dietary calcium; excessive intake of phytates (found in whole grains and legumes) and oxalates (particularly abundant in spinach and beet greens which form insoluble complexes with magnesium); chronic diarrhea; and medications such as PPIs, thiazide diuretics, and calcineurin inhibitors [71].

### Renal reabsorption of magnesium

**Fig. 4:**
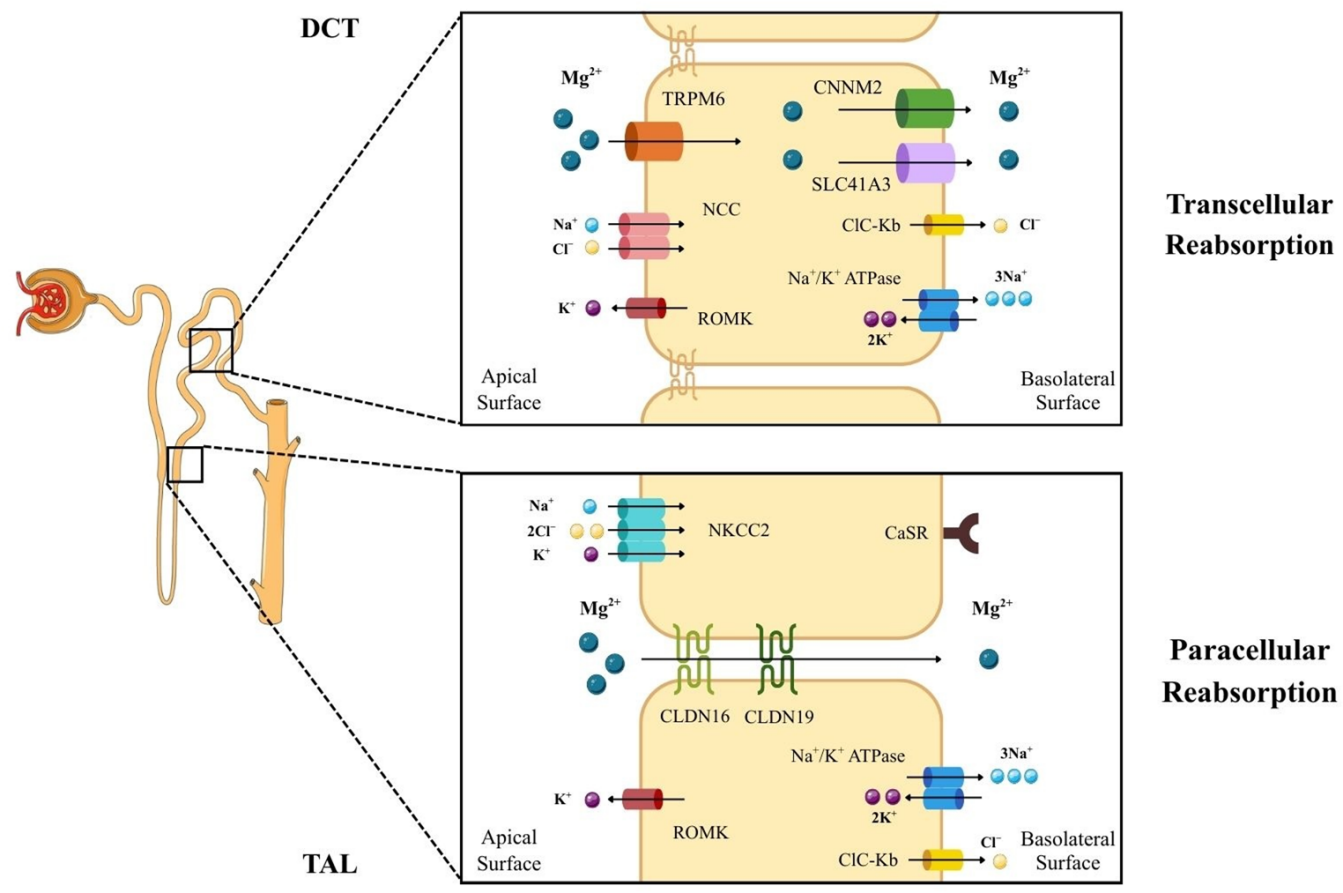
Renal magnesium reabsorption is conducted via a transcellular pathway in the DCT and via a paracellular pathway in the TAL. Abbreviations, Ca2+: calcium ion; CaSR: calcium-sensing receptor; Cl−: chloride ion; ClC-Kb: chloride channel Kb; CLDN16: claudin-16; CLDN19: claudin-19; CNNM2: cyclin M magnesium transporter 2; DCT: distal convoluted tubule; K+: potassium ion; Mg2+: magnesium ion; Na+: sodium ion; NCC: sodium–chloride cotransporter; NKCC2: sodium–potassium–chloride cotransporter 2; ROMK: renal outer medullary potassium channel; SLC41 A3: solute carrier family 41 A3; TAL: thick ascending limb; TRPM6/7: transient receptor potential melastatin channels 6 and 7. Parts of the figure are from the free medical site http://smart.servier.com/ by Servier licensed under a Creative Commons BY 4.0 License https://creativecommons.org/licenses/by/4.0/ (accessed on 12 December 2025)

In normal renal physiology, over 95% of filtered magnesium is reabsorbed along the nephron [74]. In contrast to most ions, such as calcium and sodium, the proximal convoluted tubule (PCT) reabsorbs approximately only a small fraction (15–25%) of magnesium, largely due to water reabsorption–driven concentration gradients [75]. The thick ascending limb (TAL) of Henle’s loop constitutes the dominant site of magnesium reabsorption (50–65%), mediated by paracellular diffusion across claudin-16 (CLDN16) and claudin-19 (CLDN19) pores and driven by a lumen-positive voltage [74]. The distal convoluted tubule (DCT) recovers 5–15% of filtered magnesium through active, transcellular mechanisms involving apical TRPM6/7 channels, providing the key fine-tuning step that determines urinary excretion and overall systemic balance [76]. Similarly to the active transcellular magnesium absorption in the colon, the exit step of magnesium reabsorption from the DCT into the bloodstream has not been firmly established. CNNM2 and SLC41A3 have been implicated as potential candidates for the basolateral magnesium efflux pathway [62]. Importantly, TAL magnesium handling is modulated by serum calcium. In hypercalcemia, the activation of the calcium-sensing receptor (CaSR) reduces calcium and magnesium reabsorption, thereby linking divalent cation regulation [74] **(**Fig. 4**)**. Estrogens enhance TRPM6 activity in the colon and DCT, reducing renal magnesium loss [77], while EGF modulates TRPM6 expression in the DCT through a paracrine mechanism [74].

### Skeletal storage of magnesium

Approximately 60% of total body magnesium resides in bone, where it provides both a buffering reservoir and direct contributions to skeletal physiology [32]. Notably, magnesium reduces the risk of fractures and osteoporosis during aging through bone activity regulation [78]. During bone repair, magnesium exerts a biphasic role: in the inflammatory phase, it promotes TRPM7-dependent macrophage activation and a pro-osteogenic immune microenvironment, while in later remodeling it stimulates osteogenesis and suppresses premature hydroxyapatite precipitation [79]. Beyond the skeleton, magnesium and TRPM7 signaling influence vascular smooth muscle cell phenotype, thereby modulating vascular calcification and linking mineral metabolism to cardiovascular health [80].

## Definition and epidemiology of hypomagnesemia

Normal serum magnesium concentrations in adults range between 0.6 and 1.1 mmol/L (1.46–2.67 mg/dl). Hypomagnesemia is generally defined as a serum magnesium concentration < 0.6 mmol/L [5]. Clinical manifestations usually become apparent at levels < 0.5 mmol/L, with 0.5–0.6 mmol/L indicating mild deficiency, 0.4–0.5 mmol/L moderate, and < 0.4 mmol/L severe hypomagnesemia [81]. Importantly, intracellular depletion may occur even when serum levels remain within the normal range, a condition referred to as normomagnesemic magnesium deficiency. Individuals most likely to exhibit intracellular deficiency despite normal serum magnesium include those with diabetes/insulin resistance, chronic alcohol use, long-term PPI or diuretic therapy, gastrointestinal malabsorption, and older adults. This possibility should be suspected in patients with persistent hypokalemia, unexplained hypocalcemia, frequent ventricular ectopy or arrhythmias, neuromuscular irritability, and poor glycemic control [82]. Because many patients with borderline hypomagnesemia remain asymptomatic, some experts propose raising the lower diagnostic threshold to improve sensitivity for chronic latent deficiency, though this remains debated and requires further clinical validation [83].

The prevalence of hypomagnesemia is heterogeneous and highly dependent on clinical context. In population-based studies, it is reported in 1.5%–15% of the general population [5] and in 10% of hospitalized patients [84]. Large cohort analyses reinforce this burden. NHANES data indicate that up to 48% of US adults consume less magnesium than the EAR [85], and low serum magnesium correlates with higher rates of metabolic syndrome, cardiovascular mortality, and all-cause mortality [53, 86, 87]. The European Prospective Investigation into Cancer and Nutrition (EPIC) cohorts similarly report widespread suboptimal magnesium intake in European populations [88], which in the EPIC–Norfolk cohort is associated with a higher risk of hypertension and stroke [89]. Low magnesium concentrations were linked to a higher risk of major adverse cardiovascular events (MACEs) and circulatory system disorders in a Scottish population [90]. Finally, lower serum magnesium was associated with an increased risk of developing type 2 DM (T2DM) in population-based studies [91, 92].

Several groups exhibit increased vulnerability to magnesium deficiency due to increased physiological demands or impaired homeostasis. Individuals with chronic alcohol use often develop hypomagnesemia through multiple mechanisms, including reduced intestinal absorption, inadequate dietary intake, and the direct effects of ethanol on proximal tubular handling, which collectively enhance urinary magnesium losses [25]. Older adults are similarly predisposed, owing to age-related declines in GI absorption, increased renal excretion, comorbidities, and frequent use of medications such as diuretics or PPIs. Athletes represent another at-risk group, as intense physical activity augments magnesium utilization and sweat-associated losses. Insufficient replacement may compromise ATP-dependent energy metabolism and muscle recovery [25]. Additional populations with increased requirements include pregnant and lactating women, in whom maternal–fetal transfer and expanded tissue needs elevate magnesium demands [93, 94]; patients with GI disorders such as celiac disease, inflammatory bowel disease, chronic diarrhea, or post-bariatric surgery, where malabsorption is prominent; and individuals with DM, where osmotic diuresis and intracellular magnesium depletion promote chronic deficits [95]. Finally, patients with cancer or organ transplantation frequently experience disturbances in magnesium homeostasis, partly due to disease-related metabolic alterations and the use of magnesiuric immunosuppressive or chemotherapeutic agents [74].

## Clinical manifestation of hypomagnesemia

**Fig. 5:**
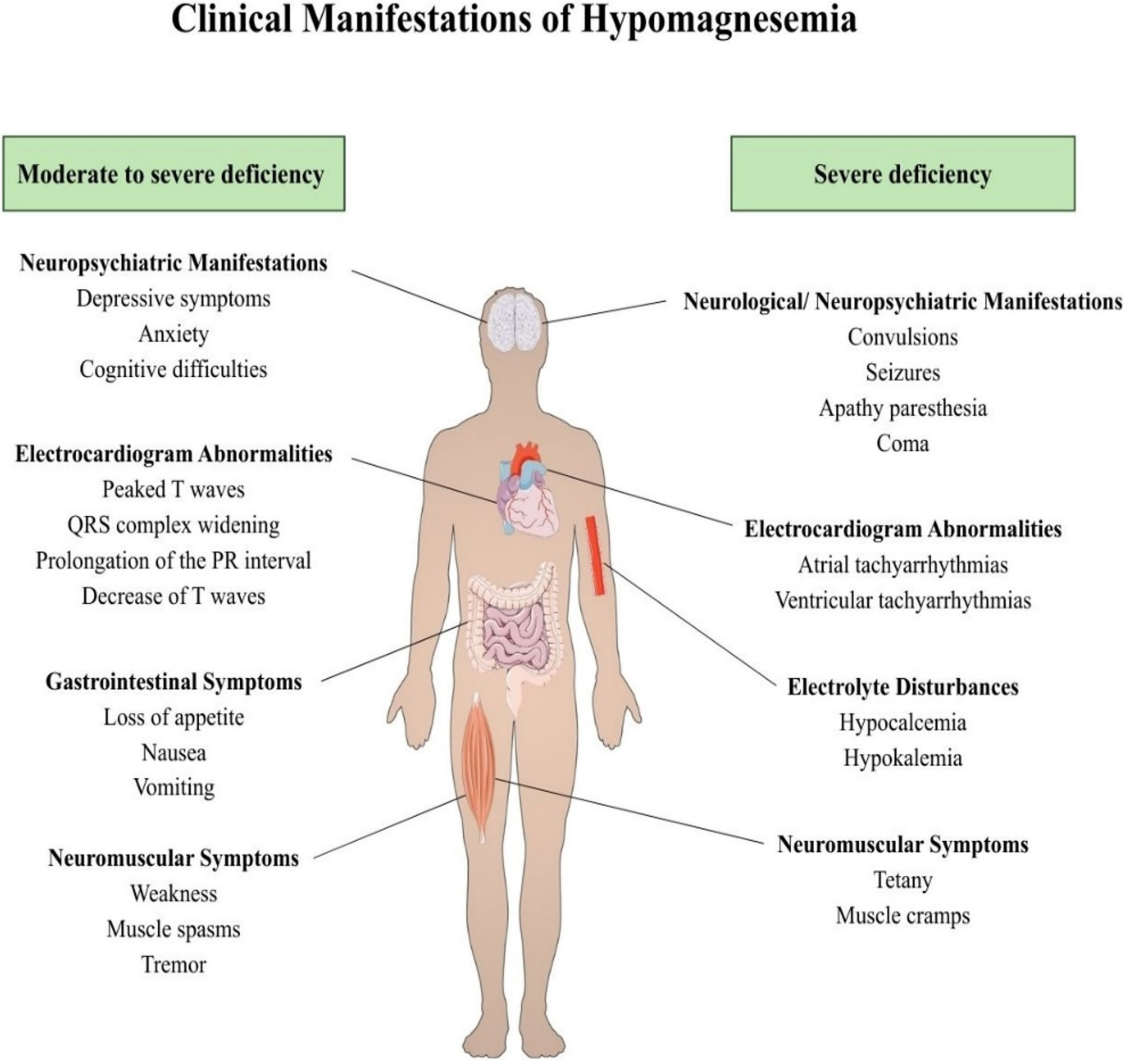
Clinical manifestation of hypomagnesemia according to severity of symptoms. Parts of the figure are from the free medical site http://smart.servier.com/ by Servier licensed under a Creative Commons BY 4.0 License https://creativecommons.org/licenses/by/4.0/ (accessed on 12 December 2025)

Hypomagnesemia is relatively common in the general population, although the majority of the affected individuals remain asymptomatic [74]. When symptomatic, its clinical spectrum ranges from mild to moderate or even severe [81]. Patients with mild or moderate magnesium deficiency most commonly present with nonspecific symptoms such as lethargy, muscle cramps, or muscle weakness. Neuromuscular irritability (including tremor, carpopedal spasm, and seizures), significant electrocardiogram abnormalities (such as peaked T waves, QRS complex widening, prolongation of the PR and QT/QTc intervals, and T wave changes), and gastrointestinal symptoms (including loss of appetite, nausea, and vomiting) are typically seen only in moderate-to-severe deficiency (serum magnesium < 0.5 mmol/L or < 1.2 mg/dL]) [9]. Beyond neuromuscular findings, neuropsychiatric manifestations may also occur, and low magnesium status has been associated with depressive symptoms, anxiety, and cognitive difficulties, reflecting the regulatory effects of magnesium on NMDA receptor activity, GABAergic signaling, and neuroinflammation [96, 97]. Severe hypomagnesemia may present with tetany, convulsions, seizures, apathy, paresthesia, and even coma, while atrial or ventricular tachyarrhythmias may also occur [9]. Importantly, hypomagnesemia often coexists with other electrolyte disorders, most notably hypocalcemia and hypokalemia, which may further complicate the clinical picture, particularly with respect to the risk of malignant arrhythmias [98] **(**Fig. 5**)**.

## Etiology of hypomagnesemia

### Insufficient Magnesium intake

Hypomagnesemia may arise from insufficient magnesium intake. Factors contributing to inadequate dietary supply include chronic alcohol consumption, modern dietary patterns dominated by processed foods and demineralized water, reduced mineral bioavailability secondary to contemporary agricultural practices, and in extreme cases, frank starvation [25]. Magnesium deficiency may also develop in settings of limited nutrient delivery, such as in individuals receiving parenteral nutrition, and it can manifest during refeeding syndrome when intracellular shifts outpace replenishment [74].

### Gastrointestinal losses

Losses from the GI tract represent another major cause of hypomagnesemia. Conditions associated with impaired absorption or excessive losses, such as acute pancreatitis with steatorrhea, malabsorptive disorders, surgical resection of the small or large intestine, and inflammatory bowel disease, have all been linked to reductions in serum magnesium levels [74].

### Inherited disorders

Inherited disorders represent an important cause of hypomagnesemia, particularly those that disrupt renal tubular handling of magnesium. Gitelman syndrome, an autosomal recessive condition affecting approximately 1 in 40,000 individuals, is characterized by loss-of-function mutations in the sodium–chloride cotransporter (NCC) encoded by the *SLC12A3* gene in the DCT, leading to reduced TRPM6 expression and impaired distal tubular magnesium reabsorption [74]. Clinically, hypomagnesemia in this syndrome presents with hypokalemia, hypocalciuria, and metabolic alkalosis [62]. Bartter syndrome type 3 (BS3), another autosomal recessive tubulopathy with a prevalence of roughly 1 in 100,000, involves mutations in the chloride channel Kb (ClC-Kb) on the basolateral membrane of the TAL, resulting in early-onset polyuria, polydipsia, and hypomagnesemia. Hypomagnesemia with secondary hypocalcemia (HSH) is likewise inherited in an autosomal recessive manner and is caused by the down-regulation of TRPM6, producing profound magnesium depletion through combined intestinal malabsorption and renal loss [74]. In familial hypomagnesemia with hypercalciuria and nephrocalcinosis (FHHNC), mutations in claudin-16 or claudin-19 disrupt paracellular magnesium and calcium transport in the TAL, leading to hypomagnesemia, hypercalciuria, and progressive nephrocalcinosis [74]. Additionally, autosomal dominant hypocalcemia due to activating mutations in the CaSR impairs renal reabsorption of both calcium and magnesium, contributing to persistent hypomagnesemia. Finally, several forms of isolated dominant or recessive hypomagnesemia have been described, reflecting heterogeneous genetic defects that ultimately result in low circulating magnesium concentrations [74].

### Medication use

Medication use is one of the most common contributors to hypomagnesemia in the general population. PPIs are particularly well recognized in this regard, with hypomagnesemia now considered a class-related adverse effect, and reported in up to 12–20% of users [99]. The risk increases with higher doses [100] being strongly associated with prolonged therapy beyond one year [40]. Mechanistically, PPIs raise luminal pH in the small intestine [101], reducing magnesium solubility and impairing passive absorption, while also significantly disrupting TRPM6-mediated active transport in the colon [66]. Reduced gut microbial diversity [38] and inhibition of the colonic H^+^/K^+^–ATPase [66], a homolog of the gastric enzyme, further alkalinize the colonic environment and suppress TRPM6 activity [102]. Impaired magnesium absorption has also been documented with agents that induce diarrhea, including laxatives and bowel preparations, select antibiotics, chemotherapeutics, colchicine, and potassium-binding resins [38].

Renal magnesium loss is another major mechanism underlying drug-induced hypomagnesemia. Loop diuretics and especially thiazides are well-established causes, acting through disruption of paracellular transport and down-regulation of TRPM6, respectively [103]. The effect is amplified when multiple nephron segments responsible for magnesium reabsorption are simultaneously inhibited, such as during combination therapy [38]. Calcineurin inhibitors similarly reduce TRPM6-dependent reabsorption in the DCT, typically producing mild but clinically significant hypomagnesemia [104]. Down-regulation of TRPM6 in the distal nephron has also been observed with epidermal growth factor receptor (EGFR) inhibitors, including cetuximab, panitumumab, and zalutumumab [105], and with mammalian target of rapamycin (mTOR) inhibitors [8]. Additional nephrotoxic agents associated with magnesium wasting include amphotericin B, which increases tubular membrane permeability and can cause overt tubular damage or necrosis; aminoglycosides, which interact with the CaSR and may provoke persistent hypomagnesemia even after discontinuation; and platinum-based chemotherapeutics such as carboplatin, oxaliplatin, and particularly cisplatin [106, 107]. Cisplatin-associated hypomagnesemia is often dose-dependent, exacerbated by GI losses, and may occur in up to 90% of patients without prophylactic supplementation, with long-term or irreversible deficits reported in some cases [38].

### COVID-19

Coronavirus disease 2019 (COVID-19) infection has been increasingly linked to disturbances in magnesium balance. Severe SARS-CoV-2–associated inflammation may precipitate a cytokine storm, which disrupts magnesium homeostasis through systemic inflammatory and metabolic effects [108]. In critically ill patients, additional factors such as respiratory alkalosis, acute kidney injury (AKI), and fluid-induced electrolyte shift further compound cation imbalances. Marked elevations in inflammatory and oxidative stress pathways, which are hallmarks of severe COVID-19, can also enhance extrarenal and renal magnesium losses, with increased urinary excretion and sweat losses frequently observed [109]. Collectively, these mechanisms place patients with severe COVID-19 at an increased risk for clinically significant hypomagnesemia.

## Multisystem disorders associated with hypomagnesemia

### Diabetes mellitus and magnesium

DM is strongly linked to altered magnesium metabolism. Reduced intracellular magnesium concentrations are frequently observed in individuals with impaired fasting glucose or insulin resistance [110, 111], and hypomagnesemia is reported in 13.5–47.7% of patients with T2DM [112, 113]. Notably, a nationwide US study of over 1.28 million veterans with T2DM has found a U-shaped association between serum magnesium levels and major cardiovascular events (MACEs), with increased risk at both low and high magnesium levels. Among patients with hypomagnesemia, prescribed oral magnesium supplementation was associated with a significantly lower risk of adverse cardiovascular outcomes, particularly in those using PPIs or thiazides [112].

The association between hypomagnesemia and DM is bidirectional. In DM, magnesium loss arises partly from impaired insulin signaling, since insulin normally enhances TRPM6-mediated magnesium reabsorption. Besides, glomerular hyperfiltration, increased urinary flow, and autonomic neuropathy–related GI malabsorption further exacerbate magnesium depletion [74]. Conversely, magnesium plays a critical role in insulin sensitivity by modulating the tyrosine kinase activity of the insulin receptor [29]. Deficiency impairs insulin trafficking and reduces GLUT4-dependent glucose uptake in peripheral tissues, while also diminishing GLUT4 gene expression, altering hepatic gluconeogenic enzyme activity such as glucose-6-phosphatase, and promoting pro-inflammatory signaling in adipose tissue [114]. Consistent with these mechanistic links, higher magnesium intake is associated with a significantly lower risk of prediabetes [91] and T2DM [115]. Magnesium supports beta-cell pancreatic function by enabling the enzymatic machinery for insulin synthesis and secretion, maintaining appropriate calcium signaling, and promoting insulin sensitivity in target tissues [116]. However, based on meta-analytic data, routine magnesium supplementation cannot be advised as a strategy to improve glycemic control in patients with T2DM [117].

### Cardiovascular disorders and magnesium

Beyond glycemic regulation, magnesium status has important implications for cardiovascular health. Both serum and dietary magnesium are inversely associated with hypertension [118, 119]. Importantly, a meta-analysis of 38 Randomized Controlled Trials (RCTs) involving 2,709 participants showed that magnesium supplementation modestly reduced systolic and diastolic blood pressure. Greater blood pressure reductions were observed in hypertensive individuals, especially those on antihypertensive therapy, and in participants with hypomagnesemia, while effects in normotensive subjects were not significant [120]. Magnesium counterbalances vasoconstrictor pathways involving bradykinin, angiotensin II, serotonin and calcium [121], and contributes to vascular homeostasis through antioxidant and anti-inflammatory actions [114]. Collectively, these findings underscore the central role of magnesium in metabolic and vascular physiology, and highlight its relevance in the prevention and management of cardiometabolic disease.

Hypomagnesemia is consistently linked to a higher risk of cardiovascular disease (CVD), including coronary heart disease and heart failure [122], as well as atrial fibrillation especially in peritoneal dialysis patients [123]. Low serum magnesium is a significant risk factor for both all-cause and cardiovascular mortality in patients receiving kidney replacement therapy, particularly those on hemodialysis [124]. Importantly, it independently predicts all-cause mortality in individuals with CKD and end-stage renal disease, as well as both cardiovascular and overall mortality in patients on maintenance hemodialysis [125].

Mechanistically, magnesium deficiency predisposes to coronary vasospasm, which can precipitate myocardial infarction, and reduces endothelial NO bioavailability, thereby promoting thrombosis and atherosclerotic progression [126]. Additional contributors include enhanced phagocyte activation and inflammatory signaling, along with neuroendocrine and renin–angiotensin–mediated stress responses that further exacerbate CVD risk [127].

### Neurological and mental disorders, and magnesium

Magnesium plays a critical role in neurophysiology. As a natural blocker of the NMDA receptor at resting membrane potential [37], magnesium limits excitatory neurotransmission while its deficiency reduces this inhibition, amplifying calcium influx, oxidative stress, neuronal injury, and ultimately neuronal cell death [32]. These mechanisms have been implicated in epilepsy [128], preeclampsia and eclampsia [29], anxiety disorders, and Alzheimer’s disease (AD) [129]. Notably, clinical studies have also shown lower magnesium levels in individuals with migraine and cluster headaches [29], while inadequate magnesium intake and low serum magnesium have been associated with depressive symptoms [130, 131]. Interestingly, a recent meta-analysis of cohort studies found a consistent U-shaped association between serum magnesium and risk of all-cause dementia and cognitive impairment, with hypomagnesemia (< 0.75 mmol/L) associated with increased risk (pooled HR 1.43, 95% CI 1.05–1.93) compared to optimal levels (~ 0.85 mmol/L) [132]. Another meta-analysis specifically in AD showed that circulating magnesium levels are significantly lower in patients compared to healthy controls, supporting hypomagnesemia as a risk factor for cognitive impairment [133]. Nevertheless, these findings derive largely from cohort data and should be interpreted as associations. Randomized evidence on prevention of dementia or neurodegeneration with magnesium repletion is currently limited and inconsistent.

With respect to anxiety and depressive disorders, meta-analyses have reported an association between hypomagnesemia and depression, but not consistently with anxiety. In particular, a meta-analysis of observational studies including 19,137 participants found that individuals with hypomagnesemia have a higher risk of depression (pooled RR 1.34, 95% CI 1.01–1.79) compared to those with normal magnesium levels, although sensitivity analyses showed marginal significance when restricted to cohort and case-control studies [131]. A recent dose-response meta-analysis of epidemiologic studies reported that higher dietary magnesium intake is associated with a lower risk of depression, with each 100 mg/day increment linked to a 7% reduction in risk (RR 0.93, 95% CI 0.90–0.96) [134]. On the contrary, no meta-analysis has established a consistent association between hypomagnesemia and anxiety disorders [97, 135].

### Miscellaneous disorders and magnesium

In the skeletal system, magnesium deficiency contributes to osteoporosis by enhancing inflammatory cytokine production and stimulating osteoclast differentiation and bone resorption [136]. Notably, a meta-analysis of 2,776 postmenopausal women found that those with osteoporosis had significantly lower serum magnesium concentrations compared to controls [137]. Additionally, a meta-analysis in adults aged more than 60 years found a significant positive association between higher magnesium intake and increased hip bone mineral density, supporting a protective role for magnesium against osteoporosis [138].

With respect to cancer, hypomagnesemia is more frequently observed in cancer patients, often due to cancer therapies (e.g. platinum-based chemotherapy, EGFR inhibitors), and is associated with increased morbidity, worse prognosis, and impaired antioxidant and immune defenses [139]. Epidemiologic and prospective studies consistently show that low dietary or serum magnesium is associated with higher risk of incident cancer and cancer mortality, particularly for colorectal, hepatocellular, and gastric cancers [140–143]. However, some evidence suggests a U-shaped relationship with both low and high plasma magnesium concentrations being associated with increased risk of incident cancer [144]. Mechanistically, magnesium deficiency may increase susceptibility to oncogenesis via oxidative stress and immune dysfunction as well as impaired genomic stability and defective DNA repair [145]. Importantly, most data linking magnesium status to cancer risk are observational and therefore associative; causality remains uncertain and RCT evidence on cancer endpoints is limited.

Immune dysregulation, including reduced T and B cell activity, and vitamin D activation, is also a recognized consequence of magnesium deficiency. Low magnesium, often in combination with imbalances in calcium and phosphate, may compromise innate and adaptive immune responses, increasing vulnerability to viral infections, including COVID-19 [146]. The interaction between magnesium status and COVID-19 is bidirectional, as infection may drive magnesium depletion, whereas pre-existing deficiency impairs antiviral signaling and cytokine regulation [109, 147]. Notably, a nationwide US cohort study demonstrated that populations residing in low-magnesium areas had a significantly higher risk of COVID-19 infection, with the effect most pronounced in children, older adults, and Black populations [148]. In hospitalized COVID-19 patients, low serum magnesium at admission independently predicted increased in-hospital and long-term mortality, longer hospital stays, and higher incidence of long COVID symptoms, even after adjustment for confounders (HR 1.29–1.57) [149, 150]. A low magnesium-to-calcium ratio was also strongly associated with mortality in severe COVID-19 (OR up to 6.93) [151].

Overall, these findings emphasize the central role of magnesium in maintaining cardiovascular, neurological, musculoskeletal, and immune homeostasis.

## Electrolyte disorders associated with hypomagnesemia

Magnesium plays a central role in maintaining potassium and calcium homeostasis, and disturbances in magnesium balance frequently coexist with abnormalities in these electrolytes [76]. Combined hypomagnesemia, hypokalemia, and hypocalcemia are clinically significant, as this triad is associated with prolonged hospitalization, increased duration of mechanical ventilation, longer intensive care unit (ICU) stays, and higher mortality [152]. The interdependence between magnesium and potassium is well established. Magnesium deficiency impairs the Na^+^–K^+^–ATPase, promoting renal potassium losses [153, 154], while loss of magnesium-mediated inhibition of the renal outer medullary potassium channel (ROMK) in the TAL further augments potassium wasting [154]. The coexistence of hypomagnesemia and hypokalemia markedly increases the risk of cardiac arrhythmias, particularly in the setting of GI losses, chronic alcohol use, or diuretic therapy [82].

Magnesium deficiency also disrupts calcium and phosphate homeostasis. Renal magnesium wasting is frequently accompanied by hypophosphatemia [155], while magnesium depletion disturbs the calcium–phosphorus balance, potentially increasing susceptibility to hypocalcemia, including in long COVID patients [156]. The relationship between magnesium and calcium is reciprocal. Hypercalcemia may induce hypomagnesemia through CaSR activation, which diminishes paracellular reabsorption of both ions [74], whereas low magnesium impairs CaSR-mediated PTH secretion, leading to reduced PTH levels and secondary hypocalcemia [81]. Interestingly, magnesium is also required for the enzymatic activation of vitamin D. Therefore, inadequate magnesium prevents the formation of active vitamin D metabolites, contributing to refractory hypocalcemia [157, 158]. Severe combined magnesium and calcium deficiency may manifest as vitamin D–resistant rickets. Given these complex interactions, magnesium imbalance should be systematically considered whenever unexplained potassium, calcium or phosphate abnormalities are encountered in clinical practice [81].

## Laboratory assessment of magnesium

The assessment of magnesium status relies on the determination of total or ionized magnesium across various biological samples using multiple analytical methods. Blood remains the most accessible matrix, with serum preferred over plasma because anticoagulants in plasma tubes, such as ethylenediaminetetraacetic acid (EDTA), citrate, and oxalate, may chelate magnesium or displace it from albumin, potentially altering results [25, 159]. Magnesium in serum can be quantified as total or ionized magnesium. Ionized magnesium reflects the biologically active fraction; however, its determination requires specialized equipment, which is not widely available. Moreover, ionized magnesium has uncertain diagnostic advantage over total magnesium [160]. Thus, total serum magnesium, which includes both the free ion and the fraction bound to proteins or complexed with anions, remains the most practical and widely used clinical measure [40].

Another easily obtainable specimen for magnesium assessment is urine. However, urinary magnesium excretion is influenced by the circadian rhythm, being higher in the evening, and is further affected by hormonal and pharmacological factors [25]. Consequently, urinary magnesium concentrations correlate poorly with overall magnesium status and are typically measured alongside serum levels, primarily to help identify the underlying cause of hypomagnesemia [161].

Although serum magnesium is the most common parameter measured [40], it represents only a small fraction of total body magnesium, which resides mainly in the intracellular space, and may thus not detect intracellular depletion [121]. Subclinical deficiency may occur despite normal serum levels [38]. Intracellular magnesium can be assessed in erythrocytes, though its clinical value is debated and may be limited to chronic deficiency states [114]. Muscle biopsy provides the most accurate reflection of intracellular stores, particularly in cardiac and skeletal muscle; nevertheless, its invasiveness, cost, and technical expertise restrict routine use. It is possible to evaluate intracellular magnesium without invasive sampling by employing sublingual epithelial cell mineral-electrolyte analysis or hair mineral analysis [162].

Pre-analytical factors may significantly influence laboratory results. Short bursts of maximal exercise or vegetarian diets could increase serum magnesium [163, 164], whereas endurance exercise, hypoalbuminemia, or late pregnancy may falsely lower magnesium values [38]. Free magnesium assays may be affected by silicones and thiocyanate in cigarette smoke, and nutrient-rich supplements in lipids, proteins, carbohydrates, various amino acids, trace elements, and vitamins [165]. EDTA contamination should be suspected when hypocalcemia, hyperkalemia, low zinc, and low alkaline phosphatase (ALP) occur simultaneously [166]. Hemolysis is a major cause of falsely elevated magnesium due to its release from intracellular stores [167], particularly in hemolytic disorders or during erythropoietin therapy [38]. Finally, sampling near infusion sites may cause spurious hypo- or hypermagnesemia depending on dilution or magnesium-containing fluids [81].

Routine laboratory quantification of total magnesium most commonly employs automated colorimetric dye-binding methods, such as xylidyl blue and calmagite assays, because they are practical and economical for high-throughput clinical use [168]. However, it is well documented that these methods are affected by pre-analytical interferences, e.g. hemolysis introduces additional magnesium from erythrocytes, while lipemia and icterus can alter absorbance readings and bias results [168]. Enzymatic and atomic absorption methods are less susceptible to interferences but are less commonly used in routine practice. Atomic absorption spectroscopy (AAS) offers superior accuracy but is impractical for routine use due to cost and technical requirements [169]. Other methods include enzymatic assays, nuclear magnetic resonance (NMR) spectroscopy for intracellular Mg^2+^–ATP quantification [170], and ion-selective electrodes (ISE). ISEs allow direct measurement of ionized free magnesium, which may be advantageous in patients with altered protein binding (e.g. hypoalbuminemia), but pre-analytical instability and lack of standardized reference ranges limit their widespread adoption [168, 171]. Additional investigational approaches include optical magnesium-specific chemosensors and fluorescent dyes [25].

Urine analysis for magnesium is primarily used to determine the underlying cause of hypomagnesemia by differentiating renal from extrarenal losses. A fractional excretion of magnesium (FEMg) > 3–4% suggests renal magnesium wasting, whereas lower values indicate insufficient intake or GI losses [38]. Twenty-four-hour magnesium excretion > 10–30 mg/24 h supports renal wasting, while < 10 mg/24 h suggests extrarenal depletion [159]. Similarly, the urinary magnesium-to-creatinine ratio (reference values in adults: 0.04–0.12 mg/mg creatinine) provides an alternative screening approach in random urine samples, reflecting renal loss at higher values and extrarenal deficiency at lower values [172]. When extrarenal magnesium depletion is suspected, further evaluation can be performed using a parenteral magnesium loading test. In individuals with sufficient magnesium stores, up to 80% of the administered magnesium is excreted, corresponding to a 24-hour retention rate of approximately 14%. In contrast, magnesium-depleted individuals typically retain around 85% of the administered dose [173]. The performance of this test is not appropriate in the context of renal magnesium wasting disorders and requires caution when kidney function is compromised [38]. Table 1 summarizes commonly used biological specimens for evaluating magnesium status, outlining what each sample type reflects, its major strengths and limitations, as well as its appropriate clinical applications.

**Table 1:** Sample types for magnesium assessment: physiological meaning, advantages, limitations, and clinical use

Sample Type	What It Reflects	Advantages	Limitations	Clinical Utility
Serum (total Mg)	Extracellular magnesium pool	Widely available; inexpensive; standardized assays; good for trending	Poor reflection of intracellular stores; affected by albumin, hemolysis, and dilution	First-line test for suspected deficiency; monitoring during supplementation
Serum (ionized Mg)	Biologically active free Mg²⁺ fraction	Theoretically superior indicator of physiologic magnesium status	Requires specialized equipment; limited availability; uncertain diagnostic advantage	Adjunctive use in critical care or research settings
Plasma	Extracellular magnesium	Easy to obtain	Anticoagulants may chelate Mg and distort results	Not preferred; use only if serum unavailable
Erythrocytes (RBC Mg)	Intracellular magnesium pool	May detect chronic or subclinical deficiency	Technically demanding; variable reference ranges; not universally standardized	Occasional adjunct test when serum Mg is normal but deficiency suspected
Muscle biopsy	Gold-standard intracellular magnesium content	Most accurate measure of tissue Mg stores	Invasive, costly, requires specialized lab; impractical for routine use	Reserved for research or complex diagnostic cases
Urine (random sample)	Renal handling of magnesium at a single time point	Convenient to collect; used to calculate FEMg	Affected by circadian rhythm and hydration status	Differentiates renal vs. extrarenal Mg losses
Urine (24-hour collection)	Daily renal magnesium excretion	More accurate assessment of renal Mg wasting	Burdensome collection; risk of incomplete samples	Defines renal wasting when > 10–30 mg/24 h; assesses adequacy of stores (< 10 mg/24 h)
Magnesium loading test	Whole-body magnesium retention	Most sensitive test for total body Mg deficiency	Time-consuming; contraindicated in renal failure; requires close monitoring	Clarifies deficiency when other tests equivocal
Hair or sublingual epithelial cell analysis	Long-term Mg incorporation into peripheral tissues	Non-invasive	Limited validation; susceptibility to contamination	Research tool; not recommended for clinical diagnosis

List of Abbreviations: *FEMg* fractional excretion of magnesium, *Μg* magnesium, *Mg*^*2+*^ magnesium ion, *RBC* red blood cell.

**Fig. 6:**
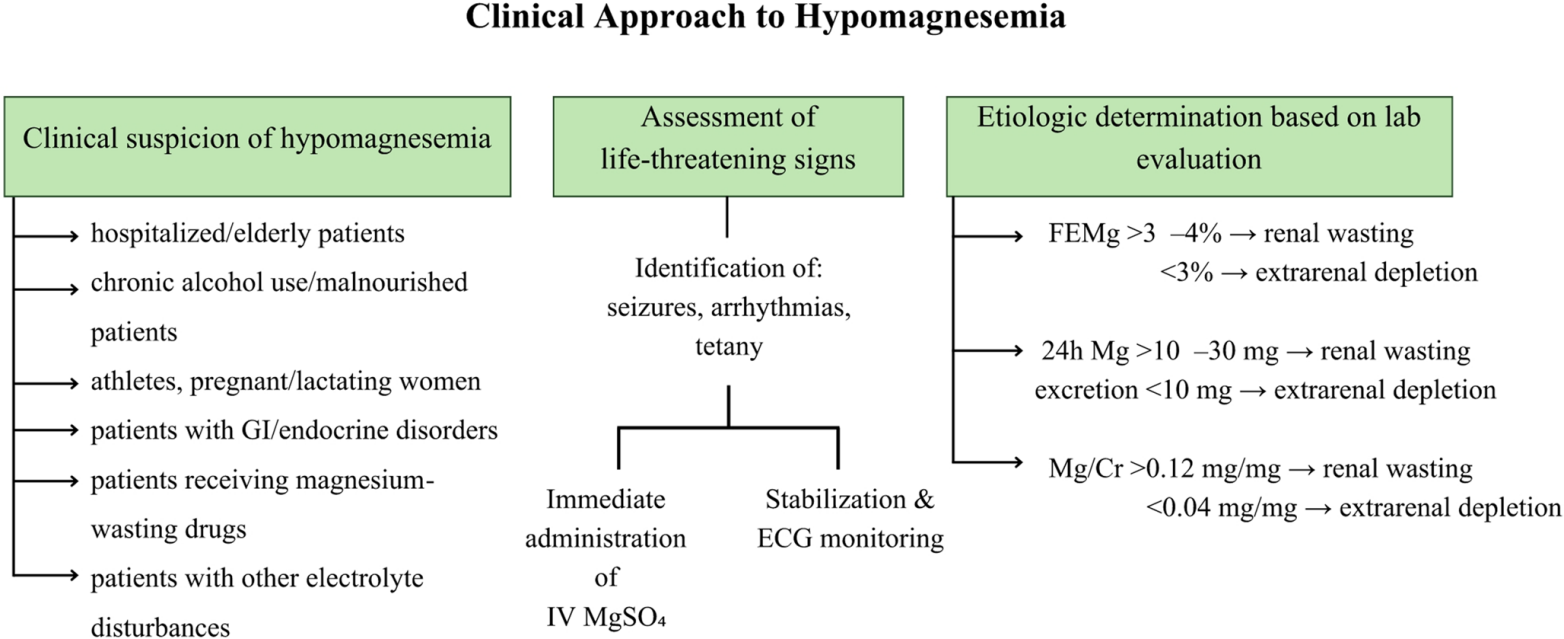
Clinical approach to hypomagnesemia based on clinical suspicion and underlying etiology. Abbreviations: *ECG* electrocardiogram, *FEMg* fractional excretion of magnesium, *GI* gastrointestinal, *IV* intravenous, *MgSO₄* magnesium sulfate

The accurate evaluation of magnesium status requires thoughtful interpretation of laboratory results depending on the clinical context. Post-analytical interpretation must account for the limitations of serum magnesium as a surrogate for total magnesium stores in the body. Normal serum magnesium (0.6–1.1 mmol/L) does not exclude intracellular deficiency, especially in chronic or subclinical states [78]. Importantly, integration with clinical context, dietary intake, and urinary magnesium excretion is recommended for comprehensive assessment [78, 174] **(**Fig. 6**)**. Testing should be pursued in patients with arrhythmias, neuromuscular symptoms, malnutrition, malabsorption, chronic alcohol use, or unexplained hypokalemia and hypocalcemia unresponsive to correction [40]. Screening is also indicated in individuals with DM, hypertension, advanced age, or long-term PPI therapy, which are frequently associated with hypomagnesemia [40]. Finally, automated laboratory demand and result management systems may improve the detection and treatment of hypomagnesemia [25].

## Treatment of hypomagnesemia

**Fig. 7:**
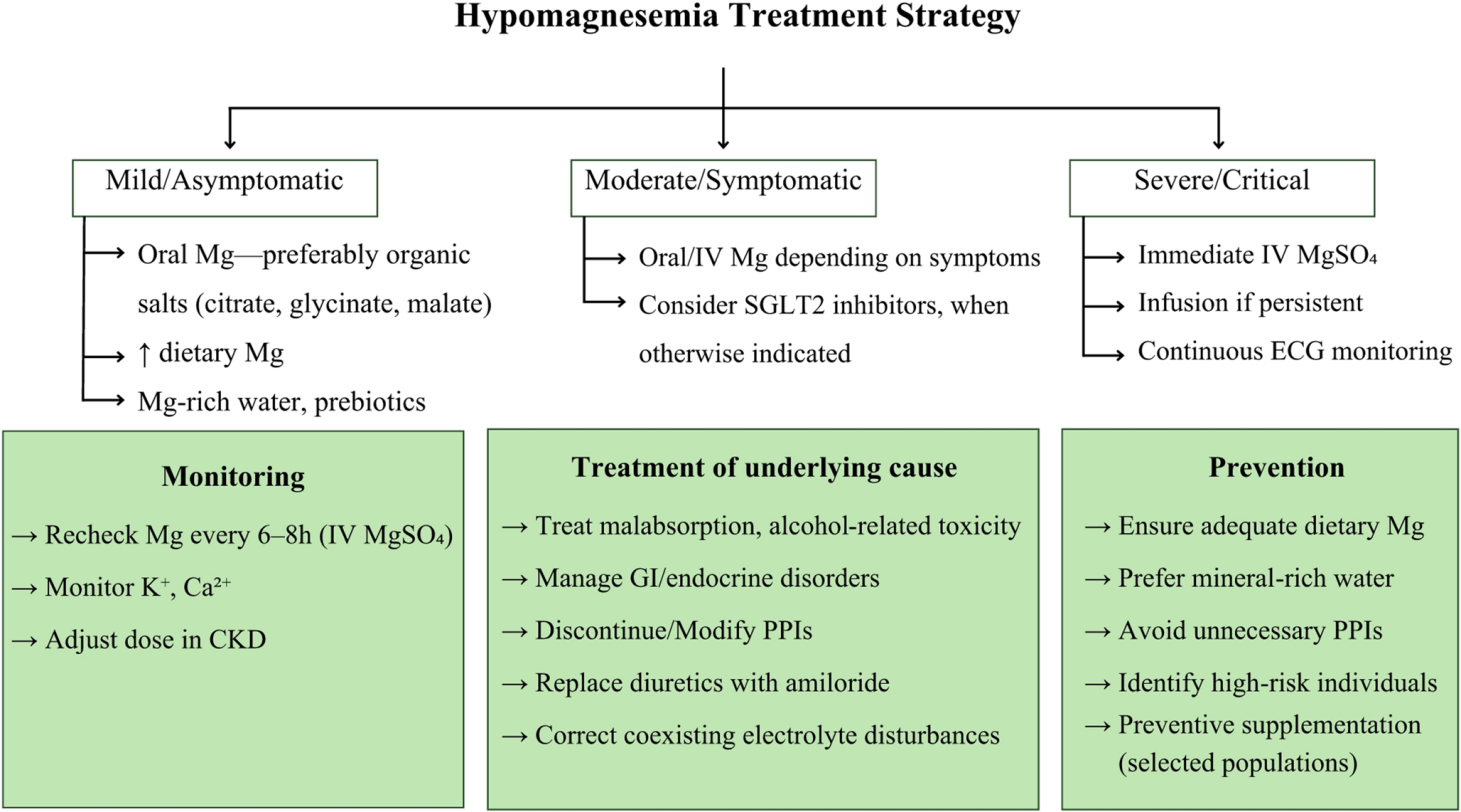
Hypomagnesemia treatment strategy. Abbreviations: *Ca2+* calcium ion, *ECG* electrocardiogram, *GI* gastrointestinal, *IV* intravenous, *K+* potassium ion, *MgSO₄* magnesium sulfate, *PPI* proton pump inhibitor, *SGLT2* sodium–glucose cotransporter 2

The therapeutic approach to hypomagnesemia is guided by renal function, hemodynamic state, and the severity of clinical manifestations [5] (Fig. 7). In patients who are asymptomatic or exhibit only mild biochemical reductions, oral replacement, i.e. typically 200–400 mg elemental magnesium daily in divided doses, is preferred, as it allows gradual correction while minimizing GI intolerance [175].

For moderate symptomatic hypomagnesemia, treatment can be administered orally or intravenously, depending on the severity of symptoms [38]. In contrast, moderate or severe deficiency, and the presence of neurological or cardiovascular complications, warrant intravenous (IV) therapy. IV magnesium is generally administered as magnesium sulfate, either as intermittent boluses for immediate relief of acute symptoms or as a continuous infusion for sustained repletion, with dosing tailored to symptom severity and renal function [38]. Patients receiving IV therapy should undergo continuous cardiac monitoring in addition to calcium and potassium level monitoring [38]. Special considerations should be given to vulnerable populations. Pregnant women with preeclampsia or eclampsia require weight-adjusted IV magnesium sulfate regimens with careful monitoring to avoid toxicity [74]. Elderly individuals and patients with chronic kidney disease (CKD) require dose reductions due to decreased magnesium clearance, while pediatric dosing must account for age-dependent renal handling and body mass [40]. Table 2 summarizes commonly used magnesium preparations for the management of hypomagnesemia, including typical adult dosing ranges, relative elemental magnesium content, pharmacokinetic and tolerability advantages, and key contraindications or precautions. For most ambulatory patients with mild-to-moderate hypomagnesemia and preserved kidney function, first-line oral options include organic magnesium salts, such as magnesium citrate, glycinate, lactate, and aspartate (typically 200–400 mg elemental Mg/day in divided doses). Intravenous magnesium sulfate is preferred for severe deficiency or when there are seizures, tetany, significant arrhythmias, hemodynamic instability, or inability to tolerate/absorb oral therapy.

**Table 2:** Magnesium supplementation: formulations, dosing strategies, advantages, limitations and contraindications

Formulation	Elemental Magnesium Content (by salt weight)	Typical Dose Range (elemental Mg)	Advantages	Limitations/Side Effects	Contraindications/ Caution
Magnesium citrate (oral)	~16%	200–400 mg elemental Mg/day in divided doses	High oral bioavailability; well tolerated; effective for mild to moderate deficiency; commonly used as an osmotic laxative for relief of occasional constipation	May cause diarrhea at higher doses; abdominal cramping, nausea; may interact with oral medications	Use cautiously in CKD (risk of accumulation); avoid in severe renal failure
Magnesium glycinate / bisglycinate (oral)	~14-15%	100–400 mg/day	High bioavailability; Well tolerated; minimal GI upset; useful for long-term supplementation	-Higher cost; limited availability in some regions -rare diarrhea; mild GI upset	CKD precautions; caution with concurrent sedating medications; severe heart block, myasthenia gravis
Magnesium lactate (oral)	~12%	200–400 mg/day	Good bioavailability and GI tolerability compared to inorganic salts such as magnesium oxide; Good absorption; Modified-release preparations may further reduce GI side effects and improve patient adherence, especially in chronic supplementation settings such as Gitelman syndrome; less laxative effect compared with oxide or citrate	Costlier than inorganic salts; less frequent GI upset; extended-release formulations may further minimize these effects	CKD precautions; Caution is also warranted in patients with severe heart block, myasthenia gravis, or those receiving medications that may increase magnesium levels (e.g. potassium-sparing diuretics)
Magnesium malate (oral)	~12-15%	100–400 mg/day	Good bioavailability and GI tolerability compared to inorganic salts; Well absorbed; Preclinical data: ↑ tissue Mg levels (muscle and brain), and may support neuromuscular function; theoretically beneficial for fatigue syndromes	Mild GI upset; diarrhea or abdominal discomfort	Renal impairment, severe heart block, myasthenia gravis, or those receiving medications that may increase magnesium levels (e.g. potassium-sparing diuretics)
Magnesium gluconate (oral)	~5–6%	100–400 mg/day	Good safety profile; less GI upset	Lower elemental Mg concentration; requires multiple tablets	Renal impairment, severe heart block, myasthenia gravis, or those receiving medications that may increase magnesium levels (e.g. potassium-sparing diuretics)
Magnesium chloride (oral)	~12%	100–400 mg/day	Better tolerated than sulfate orally; reasonable absorption; suitable for both acute and chronic supplementation; continuous-release formulations may further improve gastrointestinal tolerance and absorption	Can cause mild diarrhea (dose-dependent); strong taste in liquid form	Avoid in severe renal impairment, severe heart block, myasthenia gravis
Magnesium oxide (oral)	~60%	400–800 mg/day	High elemental magnesium; inexpensive; first-line option for chronic idiopathic constipation due to its efficacy, tolerability, and availability	Poor bioavailability due to low water solubility, resulting in less efficient absorption; high rate of diarrhea and GI distress	Avoid or used with caution in severe renal impairment (eGFR <20 mL/min/1.73 m²), severe heart block, myasthenia gravis
Magnesium carbonate (oral)	~25–29%	100–400 mg daily	Moderate Mg content; widely available and inexpensive; sometimes used as an antacid	Low bioavailability; GI upset; short-term supplementation may not reliably increase ionized Mg levels.	Avoid in severe renal impairment, severe heart block, myasthenia gravis
Magnesium acetyl taurate (oral)	~8–9%	100–400 mg daily	Good CNS penetration; ↑ bioavailability and efficient tissue penetration; rapid absorption; potential anxiolytic effects observed in animal models	Diarrhea (dose-dependent), abdominal discomfort	Caution in renal impairment, severe heart block, myasthenia gravis
Magnesium aspartate (oral)	~7-10%	100–400 mg daily	Good bioavailability; GI tolerability; suitable for chronic supplementation	GI upset, including mild diarrhea and abdominal discomfort (dose-dependent)	Caution in renal impairment, severe heart block, myasthenia gravis
Magnesium Pyroglutamate / pidolate (oral)	8-9%	200–400 mg daily	Good bioavailability; demonstrated benefit in migraine; potential utility in other neurological conditions; efficient intracellular penetration, especially in the brain	-Higher cost; diarrhea and GI distress in higher doses	Caution in renal impairment, severe heart block, myasthenia gravis
Magnesium threonate	~8%	100–400 mg daily	Good bioavailability; notable for its ability to cross the blood-brain barrier; potential cognitive and neuroprotective effects	- GI upset, including diarrhea and abdominal discomfort (dose-dependent)	Caution in renal impairment, severe heart block, myasthenia gravis
Magnesium sulfate (i.v. infusion)	~9.8%	1–2 g/hour or continuous infusion depending on severity	Allows controlled repletion; preferred in ICU settings; immediate anticonvulsant action in eclampsia	Requires serial Mg measurements; risk of overcorrection; dose-dependent effects: flushing, sweating, hypotension, bradycardia, CNS depression, loss of deep tendon reflexes, respiratory depression, and cardiac arrhythmias. Toxicity risk increases with rapid infusion or renal impairment. Serum magnesium >4 mEq/L reduces reflexes; >10 mEq/L may cause respiratory paralysis; >12 mEq/L may be fatal.	Contraindicated in severe renal failure unless dialysis available; caution in myasthenia gravis, heart block, and respiratory insufficiency; Avoid rapid infusion; always dilute to ≤20% for IV use; Injectable calcium should be available to treat magnesium toxicity.
Magnesium sulfate (i.v. bolus)	~9.8%	1–2 g MgSO₄ over 15–60 min (acute cases: seizures, arrhythmias); typical dose range: 1–5 g magnesium sulfate (98–490 mg elemental magnesium) per dose, depending on indication and severity.	Rapid correction; essential for severe or symptomatic hypomagnesemia	Requires monitoring for toxicity (hypotension, bradycardia, respiratory depression)	Same as above
Magnesium sulfate (i.m.)	~9.8%	1–5 g IM, divided doses	Alternative when i.v. access unavailable	Painful injection; erratic absorption; flushing, hypotension, bradycardia, CNS depression, loss of deep tendon reflexes, respiratory depression, and cardiac arrhythmias at ↑ serum Mg levels.	Same as above
Magnesium-containing laxatives or antacids (Mg hydroxide, carbonate)	Variable	Highly variable	Useful for constipation or dyspepsia with coexisting mild deficiency	GI cramping, diarrhea; risk of hypermagnesemia with chronic use	Avoid in CKD; avoid with concurrent Mg supplements

Data in this Table derived from literature used in the manuscript and FDA documents. List of Abbreviations: *CKD* chronic kidney disease, *GI* gastrointestinal, *ICU* intensive care unit, *IM* intramuscular, *IV* intravenous, *Mg* magnesium, *MgSO₄* magnesium sulfate

Dietary modification is important. Increasing consumption of magnesium-rich foods supports physiological repletion [74], while mineral-rich water and prebiotic fibers such as inulin may further enhance intestinal absorption, especially in individuals with subclinical deficiency [74, 176]. Oral supplementation can be provided using organic or inorganic magnesium salts. Organic salts (citrate, gluconate, lactate, aspartate) demonstrate superior bioavailability compared with inorganic forms (chloride, sulfate) [177]. Magnesium chloride is preferred for oral use due to better tolerance, whereas magnesium sulfate is the standard formulation for parenteral administration [74]. Parenteral supplementation with magnesium sulfate is indicated in neurological and cardiovascular symptomatology, including seizures, tetany, and arrhythmias, respectively [38].

Treatment should also address underlying etiologies. Current malabsorption or alcohol-related toxicity should be addressed, while magnesium depletion secondary to GI or endocrine disorders requires management of the primary condition. Coexisting electrolyte disturbances in patients with hypomagnesemia should be corrected according to standard treatment protocols [3]. PPIs associated with dose-dependent hypomagnesemia may need to be discontinued or replaced with alternative therapies [176]. In cases of renal magnesium wasting, pharmacologic interventions can reduce urinary losses. Amiloride, an epithelial sodium channel (ENaC) inhibitor, hyperpolarizes DCT cells to facilitate TRPM6/7-mediated magnesium reabsorption and is effective in diuretic-induced losses as well as Gitelman or Bartter syndromes [178]. Additionally, in patients with symptomatic hypomagnesemia, SGLT2 inhibitors may reduce renal magnesium loss and raise serum magnesium concentrations primarily by improving insulin sensitivity and enhancing TRPM6-mediated magnesium reabsorption [38]. Prevention of hypomagnesemia requires identification of high-risk individuals and guidance on adequate dietary magnesium intake, including consumption of mineral-rich water. Furthermore, the indiscriminate use of PPIs should be avoided.

The risks of indiscriminate supplementation include gastrointestinal intolerance and hypermagnesemia, particularly in CKD and older adults with reduced renal clearance. Several cohorts report U-shaped associations, with adverse outcomes observed at both low and high serum magnesium levels. Therefore, supplementation should be individualized and monitored, especially when eGFR is reduced or when magnesium-containing laxatives/antacids are used. Given the risk of both under- and over-replacement, serum magnesium should be monitored regularly during magnesium sulfate therapy, with levels checked every 6–8 h [179]. Excessive magnesium intake, especially in impaired renal excretion, may cause diarrhea [74], arrhythmias, hypotension, respiratory failure, or in extreme cases, fatal toxicity [29]. Magnesium therapy is contraindicated in severe renal failure unless dialysis is available, while caution is warranted when co-administered with potassium-sparing diuretics or glucagon due to the risk of hypermagnesemia. Finally, drug–nutrient interactions should also be considered, as oral magnesium may reduce the absorption of antibiotics, bisphosphonates, and calcium channel blockers [29].

The latest evidence from large-scale, prospective clinical trials and meta-analyses shows that magnesium supplementation in populations with hypomagnesemia or chronic disease risk yields modest but clinically important improvements in blood pressure, glycemic control, and inflammatory biomarkers [120, 180]. In hypertensive and hypomagnesemic individuals, oral elemental magnesium at median doses of 365 mg/day for 8–24 weeks reduces systolic blood pressure by approximately 2–8 mmHg and diastolic by 2–5 mmHg, with greater effects in those with baseline deficiency or on antihypertensive therapy; however, no clear dose-response relationship has been established and optimal dosing remains individualized. In patients with T2DM, oral magnesium supplementation at doses up to 500 mg/day for 12–24 weeks modestly improves glycated hemoglobin (HbA1c) (mean reduction 0.5–0.7%) and fasting glucose, supporting its adjunctive role in glycemic management, though current evidence does not support formal recommendations for routine use [180]. In addition, meta-analyses have shown that magnesium supplementation significantly lowers serum C-reactive protein (CRP) and increases NO, indicating anti-inflammatory and endothelial benefits [181]. Long-term outcomes from observational studies and umbrella reviews suggest that higher magnesium intake is associated with reduced risk of T2DM, stroke, and cardiovascular events, but RCTs have not consistently demonstrated reductions in hard endpoints such as myocardial infarction or mortality [182]. Thus, while long-term cardiovascular outcomes appear favorable in observational cohorts, definitive causal inference awaits adequately powered RCTs with hard cardiovascular endpoints.

Moreover, magnesium supplementation has been shown to lower hospitalization risk in pregnant women and to reduce both the frequency and severity of migraine episodes [182]. In CKD, magnesium supplementation may slow disease progression and reduce vascular calcification, but dosing must be carefully titrated to avoid hypermagnesemia, especially in advanced stages [183, 184]. Finally, meta-analytical data based on RCTs have shown that magnesium supplementation significantly reduces depression scores in adults with depressive disorder [185].

Moreover, magnesium supplementation has been shown to lower hospitalization risk in pregnant women and to reduce both the frequency and severity of migraine episodes [182]. In CKD, magnesium supplementation may slow disease progression and reduce vascular calcification, but dosing must be carefully titrated to avoid hypermagnesemia, especially in advanced stages [183, 184]. Finally, meta-analytical data based on RCTs have shown that magnesium supplementation significantly reduces depression scores in adults with depressive disorder [185] Overall, magnesium repletion is safe and beneficial in populations with documented deficiency or increased cardiometabolic risk, but optimal dosing regimens and long-term impact on major clinical outcomes require further investigation in ongoing and future trials [186].

Future directions in hypomagnesemia research emphasize improved diagnostic strategies, mechanistic understanding, and novel nutritional and pharmacologic interventions. Recent clinical trials and mechanistic studies have highlighted the limitations of serum magnesium as a biomarker, given its poor correlation with total body and intracellular magnesium stores. Future work is focused on developing more sensitive and specific diagnostic tools, including intracellular and functional biomarkers [186].

Advances in understanding renal and intestinal magnesium transport biology, such as the roles of TRPM6/7 channels, claudins, and CNNM transporters, have clarified the genetic and acquired mechanisms underlying hypomagnesemia, with implications for targeted therapies [187]. New mechanistic insights reveal that hypomagnesemia contributes to mitochondrial dysfunction, oxidative stress, and inflammation, mediated in part by TRPM7 kinase activity, suggesting that future therapies may include TRPM7 kinase inhibitors in addition to magnesium supplementation [188].

Updated clinical guidelines and reviews underscore the need for individualized treatment approaches, with oral organic magnesium salts preferred for mild deficiency and IV magnesium sulfate for severe or symptomatic cases. However, there remains a lack of consensus and standardized protocols for magnesium replacement, particularly in special populations such as those with CKD, DM, or drug-induced hypomagnesemia [38]. Emerging nutritional interventions include the use of prebiotic dietary fibers (e.g., inulin), while SGLT2 inhibitors may be used to enhance magnesium absorption and repletion, particularly in patients with PPI-induced or diabetic hypomagnesemia. Food fortification, personalized nutrition strategies, and dietary patterns, such as Mediterranean and plant-forward diets, are also being explored to address widespread suboptimal intake at the population level [186]. Further research is needed to determine whether biomarkers of intracellular magnesium status, and metabolomic signatures or microbiome profiles that can reliably predict deficiency or therapeutic responsiveness.

Key gaps include the need for large-scale, prospective studies and RCTs to define optimal supplementation regimens, duration and dosing strategies, long-term outcomes, and the impact of magnesium repletion on chronic disease risk and hard outcomes such as cardiovascular events, hospitalization rates or cognitive decline. There is also a need for greater awareness and routine assessment of magnesium status in clinical practice, as highlighted by recent clinical guidelines [189].

## Conclusion

 Magnesium is an important determinant of cellular homeostasis, energy metabolism, neuromuscular transmission, cardiovascular function, bone integrity, and immune regulation. Despite its physiological importance, hypomagnesemia remains under-recognized in clinical practice, owing in part to the limitations of serum magnesium as a biomarker and the frequent absence of early clinical symptoms. The increasing prevalence of overt and subclinical magnesium deficiency is driven by modern dietary patterns, chronic disease burden, medication use, and environmental factors such as mineral-depleted water.

Current evidence demonstrates that restoring magnesium status through dietary modification, oral supplementation with well-absorbed organic salts, or targeted pharmacologic interventions, can improve glycemic control, blood pressure, endothelial function, inflammatory markers, and neuromuscular symptoms in affected individuals. Recent advances in molecular biology have elucidated the roles of renal and intestinal magnesium transporters, offering new therapeutic targets and informing the use of agents such as SGLT2 inhibitors and prebiotic fibers for select populations.

Nonetheless, important gaps remain regarding the optimal assessment and management of hypomagnesemia, optimal dosing strategies, monitoring protocols, and long-term outcomes of magnesium repletion, particularly in vulnerable groups such as patients with CKD, DM, older adults or those receiving PPIs or chemotherapy.

Future research should prioritize the development of more sensitive magnesium biomarkers, robust clinical trials evaluating hard endpoints, nutrition-focused interventions aimed at population-wide prevention as well as individualized supplementation strategies. Improving clinical awareness and integrating magnesium assessment into routine care may represent a cost-effective strategy to mitigate the significant multisystem burden of hypomagnesemia.

## Key references

Papers of particular interest, published recently, have been highlighted as:

- Pitliya A, Vasudevan SS, Batra V, Patel MB, Desai A, Nethagani S, et al. Global prevalence of hypomagnesemia in type 2 diabetes mellitus - a comprehensive systematic review and meta-analysis of observational studies. Endocrine. 2024; 84(3):842 − 51.
  - Findings from this meta-analysis indicate that hypomagnesemia represents a frequent and clinically relevant disturbance in patients with type 2 diabetes mellitus.
- Argeros Z, Xu X, Bhandari B, Harris K, Touyz RM, Schutte AE. Magnesium Supplementation and Blood Pressure: A Systematic Review and Meta-Analysis of Randomized Controlled Trials. Hypertension. 2025; 82(11):1844-56.s
  - This meta-analysis indicates a potential blood pressure–lowering benefit of magnesium in hypertensive populations with hypomagnesemia.
- Chen F, Wang J, Cheng Y, Li R, Wang Y, Chen Y, et al. Magnesium and Cognitive Health in Adults: A Systematic Review and Meta-Analysis. Adv Nutr. 2024; 15(8):100272.
  - Cohort studies showed a consistent U-shaped relationship between serum magnesium levels and both all-cause dementia and cognitive impairment, indicating an optimal concentration of approximately 0.85 mmol/L.
- Asbaghi O, Moradi S, Kashkooli S, Zobeiri M, Nezamoleslami S, Hojjati Kermani MA, et al. The effects of oral magnesium supplementation on glycemic control in patients with type 2 diabetes: a systematic review and dose-response meta-analysis of controlled clinical trials. Br J Nutr. 2022; 128(12):2363-72.
  - Oral magnesium supplementation may exert a beneficial effect on glycemic control in patients with type 2 diabetes mellitus.
- Veronese N, Demurtas J, Pesolillo G, Celotto S, Barnini T, Calusi G, et al. Magnesium and health outcomes: an umbrella review of systematic reviews and meta-analyses of observational and intervention studies. Eur J Nutr. 2020; 59(1):263-72.
  - Magnesium supplementation has been shown to reduce hospitalization risk during pregnancy and to lessen both the frequency and severity of migraine. In observational studies, higher magnesium intake has also been associated with a lower risk of type 2 diabetes and stroke.
- Moabedi M, Aliakbari M, Erfanian S, Milajerdi A. Magnesium supplementation beneficially affects depression in adults with depressive disorder: a systematic review and meta-analysis of randomized clinical trials. Front Psychiatry. 2023; 14:1333261.
  - Magnesium supplementation has been associated with improvements in depressive symptoms.

## Data Availability

No datasets were generated or analysed during the current study.

## Abbreviations

AAS: atomic absorption spectroscopy
AD: Alzheimer’s disease
AI: adequate intake
AKI: acute kidney injury
ALP: alkaline phosphatase
ATP: adenosine triphosphate
BS3: Bartter syndrome type 3
Ca: calcium
Ca^2+^(Ca++): calcium ion
CaSR: calcium-sensing receptor
CKD: chronic kidney disease
CGRP: calcitonin gene–related peptide
Cl^−^: chloride ion
ClC-Kb: chloride channel Kb
CLDN16: claudin-16
CLDN19: claudin-19
CNNM: cyclin M magnesium transporter
COVID-19: coronavirus disease 2019
CRP: C-reactive protein
CVD: cardiovascular disease
DASH: Dietary Approaches to Stop Hypertension
DCT: distal convoluted tubule
DM: diabetes mellitus
EAR: estimated average requirement
ECG: electrocardiogram
EDTA: ethylenediaminetetraacetic acid
EFSA: European Food Safety Authority
EGF: epidermal growth factor
EGFR: epidermal growth factor receptor
ENaC: epithelial sodium channel
EPIC: European Prospective Investigation into Cancer and Nutrition
FEMg: fractional excretion of magnesium
FGF23: fibroblast growth factor 23
FHHNC: familial hypomagnesemia with hypercalciuria and nephrocalcinosis
GABA: gamma-aminobutyric acid
GI: gastrointestinal
H^+^: hydrogen ion
HbA1c: glycated hemoglobin
HSH: hypomagnesemia with secondary hypocalcemia
ICU: intensive care unit
IL-6: interleukin-6
IM: intramuscular
ΙSE: ion-selective electrodes
IV: intravenous
K: potassium
K^+^: potassium ion
MACEs: major adverse cardiovascular events
Mg: magnesium
Mg^2+^(Mg^++^): magnesium ion
MgSO₄: magnesium sulfate
mTOR: mammalian target of rapamycin
Na: sodium
Na^+^: sodium ion
NCC: sodium–chloride cotransporter
NF-κB: nuclear factor kappa B
NHANES: National Health and Nutrition Examination Survey
NKCC2: sodium–potassium–chloride cotransporter 2
NMDA: N-methyl-D-aspartate
NMR: nuclear magnetic resonance
NO: nitric oxide
PCT: proximal convoluted tubule
PGE1: prostaglandin E1
PGI2: prostacyclin
PPI: proton pump inhibitor
PTH: parathyroid hormone
RBC: red blood cell
RCT: randomized controlled trial
RDA: recommended daily allowance
ROMK: renal outer medullary potassium channel
SCFA: short-chain fatty acid
SGLT2: sodium–glucose cotransporter 2
SLC41: Solute Carrier Family 41
T2DM: type 2 diabetes mellitus
TAL: thick ascending limb
TRPM6/7: transient receptor potential melastatin channels 6 and 7

## References

[1] Jawaid S, Shams S, Kharb D, Al-Bar OA, Zeyadi M, Al Hazmi IS, et al. Comprehensive View of Magnesium Physiology. Endocr Metab Immune Disord Drug Targets. 2025. 10.2174/0118715303364703250224053714. 40264314 10.2174/0118715303364703250224053714 PMC13559948

[2] Jahnen-Dechent W, Ketteler M. Magnesium basics. Clin Kidney J. 2012;5(Suppl 1):i3–14. 10.1093/ndtplus/sfr163. 26069819 10.1093/ndtplus/sfr163 PMC4455825

[3] Ahmed F, Mohammed A. Magnesium: the forgotten electrolyte-a review on hypomagnesemia. Med Sci. 2019. 10.3390/medsci7040056. 10.3390/medsci7040056 PMC6524065 30987399

[4] Kurstjens S, de Baaij JHF, Overmars-Bos C, van den Munckhof ICL, Garzero V, de Vries MA, et al. Increased NEFA levels reduce blood Mg(2+) in hypertriacylglycerolaemic states via direct binding of NEFA to Mg(2. Diabetologia. 2019;62(2):311–21. 10.1007/s00125-018-4771-3. 30426168 10.1007/s00125-018-4771-3 PMC6323097

[5] Gragossian A, Bashir K, Bhutta BS, Friede R, Hypomagnesemia. 2023. In: StatPearls [Internet]. Treasure Island (FL): StatPearls Publishing; 2025. PMID: 29763179. 29763179

[6] Touyz RM, de Baaij JHF, Hoenderop JGJ. Magnesium disorders. N Engl J Med. 2024;390(21):1998–2009. 10.1056/NEJMra1510603. 38838313 10.1056/NEJMra1510603

[7] Feeney KA, Hansen LL, Putker M, Olivares-Yañez C, Day J, Eades LJ, et al. Daily magnesium fluxes regulate cellular timekeeping and energy balance. Nature. 2016;532(7599):375–9. 10.1038/nature17407. 27074515 10.1038/nature17407 PMC4886825

[8] Pham PC, Pham PA, Pham SV, Pham PT, Pham PM, Pham PT. Hypomagnesemia: a clinical perspective. Int J Nephrol Renovasc Dis. 2014;7:219–30. 10.2147/ijnrd.S42054. 24966690 10.2147/IJNRD.S42054 PMC4062555

[9] Negru AG, Pastorcici A, Crisan S, Cismaru G, Popescu FG, Luca CT. The role of hypomagnesemia in cardiac arrhythmias: a clinical perspective. Biomedicines. 2022. 10.3390/biomedicines10102356. 36289616 10.3390/biomedicines10102356 PMC9598104

[10] Dalamaga M, Karmaniolas K, Nikolaidou A, Papadavid E. Hypocalcemia, hypomagnesemia, and hypokalemia following hydrofluoric acid chemical injury. J Burn Care Res. 2008;29(3):541–3. 10.1097/BCR.0b013e3181711152. 18388571 10.1097/BCR.0b013e3181711152

[11] Caspi R, Billington R, Keseler IM, Kothari A, Krummenacker M, Midford PE, et al. The MetaCyc database of metabolic pathways and enzymes - a 2019 update. Nucleic Acids Res. 2020;48(D1):D445-d53. 10.1093/nar/gkz862. 31586394 10.1093/nar/gkz862 PMC6943030

[12] Garfinkel L, Garfinkel D. Magnesium regulation of the glycolytic pathway and the enzymes involved. Magnesium. 1985;4(2–3):60–72. 2931560

[13] Thomas AP, Diggle TA, Denton RM. Sensitivity of pyruvate dehydrogenase phosphate phosphatase to magnesium ions. Similar effects of spermine and insulin. Biochem J. 1986;238(1):83–91. 10.1042/bj2380083. 3026347 10.1042/bj2380083 PMC1147100

[14] Galkin MA, Syroeshkin AV. Kinetic mechanism of ATP synthesis catalyzed by mitochondrial Fo x F1-ATPase. Biochem (Mosc). 1999;64(10):1176–85. 10561566

[15] Feng J, Wang H, Jing Z, Wang Y, Cheng Y, Wang W, et al. Role of magnesium in type 2 diabetes mellitus. Biol Trace Elem Res. 2020;196(1):74–85. 10.1007/s12011-019-01922-0. 31713111 10.1007/s12011-019-01922-0

[16] Kostov K. Effects of magnesium deficiency on mechanisms of insulin resistance in type 2 diabetes: focusing on the processes of insulin secretion and signaling. Int J Mol Sci. 2019. 10.3390/ijms20061351. 30889804 10.3390/ijms20061351 PMC6470576

[17] Wolf FI, Maier JA, Nasulewicz A, Feillet-Coudray C, Simonacci M, Mazur A, et al. Magnesium and neoplasia: from carcinogenesis to tumor growth and progression or treatment. Arch Biochem Biophys. 2007;458(1):24–32. 10.1016/j.abb.2006.02.016. 16564020 10.1016/j.abb.2006.02.016

[18] Anastassopoulou J, Theophanides T. Magnesium-DNA interactions and the possible relation of magnesium to carcinogenesis. Irradiation and free radicals. Crit Rev Oncol Hematol. 2002;42(1):79–91. 10.1016/s1040-8428(02)00006-9. 11923070 10.1016/s1040-8428(02)00006-9

[19] Yamagami R, Bingaman JL, Frankel EA, Bevilacqua PC. Cellular conditions of weakly chelated magnesium ions strongly promote RNA stability and catalysis. Nat Commun. 2018;9(1):2149. 10.1038/s41467-018-04415-1. 29858572 10.1038/s41467-018-04415-1 PMC5984629

[20] Fandilolu PM, Kamble AS, Dound AS, Sonawane KD. Role of Wybutosine and Mg(2+) ions in modulating the structure and function of tRNA(Phe): a molecular dynamics study. ACS Omega. 2019;4(25):21327–39. 10.1021/acsomega.9b02238. 31867527 10.1021/acsomega.9b02238 PMC6921629

[21] Zocchi M, Locatelli L, Zuccotti GV, Mazur A, Béchet D, Maier JA, et al. Magnesium homeostasis in myogenic differentiation—a focus on the regulation of TRPM7, MagT1 and SLC41A1 transporters. Int J Mol Sci. 2022;23(3):1658. 10.3390/ijms23031658. 35163580 10.3390/ijms23031658 PMC8836031

[22] Ferenc K, Sokal-Dembowska A, Helma K, Motyka E, Jarmakiewicz-Czaja S, Filip R. Modulation of the gut microbiota by nutrition and its relationship to epigenetics. Int J Mol Sci. 2024. 10.3390/ijms25021228. 38279228 10.3390/ijms25021228 PMC10816208

[23] Chacko SA, Sul J, Song Y, Li X, LeBlanc J, You Y, et al. Magnesium supplementation, metabolic and inflammatory markers, and global genomic and proteomic profiling: a randomized, double-blind, controlled, crossover trial in overweight individuals. Am J Clin Nutr. 2011;93(2):463–73. 10.3945/ajcn.110.002949. 21159786 10.3945/ajcn.110.002949 PMC3021435

[24] Mammoli F, Castiglioni S, Parenti S, Cappadone C, Farruggia G, Iotti S, et al. Magnesium is a key regulator of the balance between osteoclast and osteoblast differentiation in the presence of vitamin D₃. Int J Mol Sci. 2019. 10.3390/ijms20020385. 30658432 10.3390/ijms20020385 PMC6358963

[25] Fiorentini D, Cappadone C, Farruggia G, Prata C. Magnesium: biochemistry, nutrition, detection, and social impact of diseases linked to its deficiency. Nutrients. 2021. 10.3390/nu13041136. 33808247 10.3390/nu13041136 PMC8065437

[26] Lu WC, Pringa E, Chou L. Effect of magnesium on the osteogenesis of normal human osteoblasts. Magnes Res. 2017;30(2):42–52. 10.1684/mrh.2017.0422. 28869207 10.1684/mrh.2017.0422

[27] Erem S, Atfi A, Razzaque MS. Anabolic effects of vitamin D and magnesium in aging bone. J Steroid Biochem Mol Biol. 2019;193:105400. 10.1016/j.jsbmb.2019.105400. 31175968 10.1016/j.jsbmb.2019.105400

[28] Rude RK, Singer FR, Gruber HE. Skeletal and hormonal effects of magnesium deficiency. J Am Coll Nutr. 2009;28(2):131–41. 10.1080/07315724.2009.10719764. 19828898 10.1080/07315724.2009.10719764

[29] Gröber U, Schmidt J, Kisters K. Magnesium in prevention and therapy. Nutrients. 2015;7(9):8199–226. 10.3390/nu7095388. 26404370 10.3390/nu7095388 PMC4586582

[30] White RE, Hartzell HC. Effects of intracellular free magnesium on calcium current in isolated cardiac myocytes. Science. 1988;239(4841 Pt 1):778–80. 10.1126/science.2448878. 2448878 10.1126/science.2448878

[31] Iseri LT, French JH. Magnesium: nature’s physiologic calcium blocker. Am Heart J. 1984;108(1):188–93. 10.1016/0002-8703(84)90572-6. 6375330 10.1016/0002-8703(84)90572-6

[32] de Baaij JH, Hoenderop JG, Bindels RJ. Magnesium in man: implications for health and disease. Physiol Rev. 2015;95(1):1–46. 10.1152/physrev.00012.2014. 25540137 10.1152/physrev.00012.2014

[33] Severino P, Netti L, Mariani MV, Maraone A, D’Amato A, Scarpati R, et al. Prevention of cardiovascular disease: screening for magnesium deficiency. Cardiol Res Pract. 2019;2019:4874921. 10.1155/2019/4874921. 31192005 10.1155/2019/4874921 PMC6525869

[34] Güzel A, Doğan E, Türkçü G, Kuyumcu M, Kaplan İ, Çelik F, et al. Dexmedetomidine and magnesium sulfate: a good combination treatment for acute lung injury? J Invest Surg. 2019;32(4):331–42. 10.1080/08941939.2017.1422575. 29359990 10.1080/08941939.2017.1422575

[35] Steinert JR, Postlethwaite M, Jordan MD, Chernova T, Robinson SW, Forsythe ID. *NMDAR*-mediated EPSCs are maintained and accelerate in time course during maturation of mouse and rat auditory brainstem *in vitro*. J Physiol. 2010;588(Pt 3):447–63. 10.1113/jphysiol.2009.184317. 20008465 10.1113/jphysiol.2009.184317 PMC2825610

[36] Bigal ME, Walter S, Rapoport AM. Calcitonin gene-related peptide (CGRP) and migraine current understanding and state of development. Headache. 2013;53(8):1230–44. 10.1111/head.12179. 23848260 10.1111/head.12179

[37] Paoletti P, Bellone C, Zhou Q. *NMDA* receptor subunit diversity: impact on receptor properties, synaptic plasticity and disease. Nat Rev Neurosci. 2013;14(6):383–400. 10.1038/nrn3504. 23686171 10.1038/nrn3504

[38] Rosner MH, Ha N, Palmer BF, Perazella MA. Acquired disorders of hypomagnesemia. Mayo Clin Proc. 2023;98(4):581–96. 10.1016/j.mayocp.2022.12.002. 36872194 10.1016/j.mayocp.2022.12.002

[39] Institute of Medicine Standing Committee on the Scientific Evaluation of Dietary Reference I. The National Academies Collection: Reports funded by National Institutes of Health. Dietary Reference Intakes for Calcium, Phosphorus, Magnesium, Vitamin D, and Fluoride. Washington (DC): National Academies Press (US) Copyright © 1997, National Academy of Sciences.; 1997.

[40] Salinas M, López-Garrigós M, Flores E, Leiva-Salinas C. Improving diagnosis and treatment of hypomagnesemia. Clin Chem Lab Med. 2024;62(2):234–48. 10.1515/cclm-2023-0537. 37503587 10.1515/cclm-2023-0537

[41] Martínez-Ferrer A, Peris P, Reyes R, Guañabens N. [Intake of calcium, magnesium and sodium through water: health implications]. Med Clin (Barc). 2008;131(17):641–6. 10.1157/13128721. 19087789 10.1157/13128721

[42] Thippeswamy HM, Shanbhog R, Kumar MN, Prashanth SN, Smitha P. Comparison of serum calcium, magnesium, phosphate, alkaline phosphatase, and vitamin D levels in children consuming reverse osmosis, non reverse osmosis, and high fluoride drinking water. Sci Rep. 2025;15(1):10689. 10.1038/s41598-025-94758-9. 40155753 10.1038/s41598-025-94758-9 PMC11953389

[43] Almejrad L, Levon JA, Soto-Rojas AE, Tang Q, Lippert F. An investigation into the potential anticaries benefits and contributions to mineral intake of bottled water. J Am Dent Assoc. 2020;151(12):924–e3410. 10.1016/j.adaj.2020.08.023. 33228885 10.1016/j.adaj.2020.08.023

[44] Azoulay A, Garzon P, Eisenberg MJ. Comparison of the mineral content of tap water and bottled waters. J Gen Intern Med. 2001;16(3):168–75. 10.1111/j.1525-1497.2001.04189.x. 11318912 10.1111/j.1525-1497.2001.04189.x PMC1495189

[45] Helte E, Säve-Söderbergh M, Larsson SC, Åkesson A. Calcium and magnesium in drinking water and risk of myocardial infarction and stroke-a population-based cohort study. Am J Clin Nutr. 2022;116(4):1091–100. 10.1093/ajcn/nqac186. 35816459 10.1093/ajcn/nqac186 PMC9535516

[46] Rousseau S, Kyomugasho C, Celus M, Hendrickx MEG, Grauwet T. Barriers impairing mineral bioaccessibility and bioavailability in plant-based foods and the perspectives for food processing. Crit Rev Food Sci Nutr. 2020;60(5):826–43. 10.1080/10408398.2018.1552243. 30632768 10.1080/10408398.2018.1552243

[47] Lisciani S, Aguzzi A, Gabrielli P, Camilli E, Gambelli L, Marletta L, et al. Effects of household cooking on mineral composition and retention in widespread Italian vegetables. Nutrients. 2025. 10.3390/nu17030423. 39940280 10.3390/nu17030423 PMC11820475

[48] Cazzola R, Della Porta M, Manoni M, Iotti S, Pinotti L, Maier JA. Going to the roots of reduced magnesium dietary intake: a tradeoff between climate changes and sources. Heliyon. 2020;6(11):e05390. 10.1016/j.heliyon.2020.e05390. 33204877 10.1016/j.heliyon.2020.e05390 PMC7649274

[49] Omofuma OO, Fang D, Yell N, Falomo O, Liu J, Steck SE. Trends in reported calcium and magnesium intake from diet and supplements by demographic factors: National Health and Nutrition Examination Survey, 2003–2018. J Acad Nutr Diet. 2024;124(10):1288-301.e5. 10.1016/j.jand.2024.04.017. 38718857 10.1016/j.jand.2024.04.017 PMC12244524

[50] Liu J, Huang Y, Dai Q, Fulda KG, Chen S, Tao MH. Trends in magnesium intake among Hispanic adults, the National Health and Nutrition Examination Survey (NHANES) 1999–2014. Nutrients. 2019. 10.3390/nu11122867. 31766698 10.3390/nu11122867 PMC6950381

[51] Jackson SE, Smith L, Grabovac I, Haider S, Demurtas J, López-Sánchez GF, et al. Ethnic differences in magnesium intake in U.S. older adults: findings from NHANES 2005⁻2016. Nutrients. 2018. 10.3390/nu10121901. 30518025 10.3390/nu10121901 PMC6316208

[52] Han M, Zhang Y, Fang J, Sun M, Liu Q, Ma Z, et al. Associations between dietary magnesium intake and hypertension, diabetes, and hyperlipidemia. Hypertens Res. 2024;47(2):331–41. 10.1038/s41440-023-01439-z. 37821564 10.1038/s41440-023-01439-z

[53] Wang X, Zeng Z, Wang X, Zhao P, Xiong L, Liao T, et al. Magnesium depletion score and metabolic syndrome in US adults: analysis of NHANES 2003 to 2018. J Clin Endocrinol Metab. 2024;109(12):e2324–33. 10.1210/clinem/dgae075. 38366015 10.1210/clinem/dgae075 PMC11570370

[54] Gommers LMM, Ederveen THA, van der Wijst J, Overmars-Bos C, Kortman GAM, Boekhorst J, et al. Low gut microbiota diversity and dietary magnesium intake are associated with the development of PPI-induced hypomagnesemia. FACEB J. 2019;33(10):11235–46. 10.1096/fj.201900839R. 10.1096/fj.201900839R 31299175

[55] García-Legorreta A, Soriano-Pérez LA, Flores-Buendía AM, Medina-Campos ON, Noriega LG, Granados-Portillo O, et al. Effect of dietary magnesium content on intestinal microbiota of rats. Nutrients. 2020. 10.3390/nu12092889. 32971775 10.3390/nu12092889 PMC7551274

[56] Lima FDS, Santos MQD, Makiyama EN, Hoffmann C, Fock RA. The essential role of magnesium in immunity and gut health: impacts of dietary magnesium restriction on peritoneal cells and intestinal microbiome. J Trace Elem Med Biol. 2025;88:127604. 10.1016/j.jtemb.2025.127604. 39884252 10.1016/j.jtemb.2025.127604

[57] Gommers LMM, Leermakers PA, van der Wijst J, Roig SR, Adella A, van de Wal MAE, et al. Butyrate reduces cellular magnesium absorption independently of metabolic regulation in Caco-2 human colon cells. Sci Rep. 2022;12(1):18551. 10.1038/s41598-022-21683-6. 36329098 10.1038/s41598-022-21683-6 PMC9633768

[58] Del Chierico F, Trapani V, Petito V, Reddel S, Pietropaolo G, Graziani C, et al. Dietary magnesium alleviates experimental murine colitis through modulation of gut microbiota. Nutrients. 2021. 10.3390/nu13124188. 34959740 10.3390/nu13124188 PMC8707433

[59] Gibson R, Aljuraiban GS, Oude Griep LM, Vu TH, Steffen LM, Appel LJ, et al. Relationship of calcium and magnesium intakes with the dietary approaches to stop hypertension score and blood pressure: the International Study of Macro/micronutrients and Blood Pressure. J Hypertens. 2024;42(5):789–800. 10.1097/hjh.0000000000003648. 38164982 10.1097/HJH.0000000000003648 PMC10990009

[60] Coudray C, Demigné C, Rayssiguier Y. Effects of dietary fibers on magnesium absorption in animals and humans. J Nutr. 2003;133(1):1–4. 10.1093/jn/133.1.1. 12514257 10.1093/jn/133.1.1

[61] Sabatier M, Arnaud MJ, Kastenmayer P, Rytz A, Barclay DV. Meal effect on magnesium bioavailability from mineral water in healthy women. Am J Clin Nutr. 2002;75(1):65–71. 10.1093/ajcn/75.1.65. 11756061 10.1093/ajcn/75.1.65

[62] Morrison AR. Magnesium homeostasis: lessons from human genetics. Clin J Am Soc Nephrol. 2023;18(7):969–78. 10.2215/cjn.0000000000000103. 36723340 10.2215/CJN.0000000000000103 PMC10356123

[63] Zou ZG, Rios FJ, Montezano AC, Touyz RM. TRPM7, magnesium, and signaling. Int J Mol Sci. 2019. 10.3390/ijms20081877. 30995736 10.3390/ijms20081877 PMC6515203

[64] de Baaij JH, Hoenderop JG, Bindels RJ. Regulation of magnesium balance: lessons learned from human genetic disease. Clin Kidney J. 2012;5(Suppl 1):i15–24. 10.1093/ndtplus/sfr164. 26069817 10.1093/ndtplus/sfr164 PMC4455826

[65] Krug SM, Schulzke JD, Fromm M. Tight junction, selective permeability, and related diseases. Semin Cell Dev Biol. 2014;36:166–76. 10.1016/j.semcdb.2014.09.002. 25220018 10.1016/j.semcdb.2014.09.002

[66] Gommers LMM, Hoenderop JGJ, de Baaij JHF. Mechanisms of proton pump inhibitor-induced hypomagnesemia. Acta Physiol. 2022;235(4):e13846. 10.1111/apha.13846. 10.1111/apha.13846 PMC9539870 35652564

[67] Lameris AL, Huybers S, Kaukinen K, Mäkelä TH, Bindels RJ, Hoenderop JG, et al. Expression profiling of claudins in the human gastrointestinal tract in health and during inflammatory bowel disease. Scand J Gastroenterol. 2013;48(1):58–69. 10.3109/00365521.2012.741616. 23205909 10.3109/00365521.2012.741616

[68] Quamme GA. Molecular identification of ancient and modern mammalian magnesium transporters. Am J Physiol Cell Physiol. 2010;298(3):C407-29. 10.1152/ajpcell.00124.2009. 19940067 10.1152/ajpcell.00124.2009

[69] Quamme GA. Recent developments in intestinal magnesium absorption. Curr Opin Gastroenterol. 2008;24(2):230–5. 10.1097/MOG.0b013e3282f37b59. 18301276 10.1097/MOG.0b013e3282f37b59

[70] Schlingmann KP, Waldegger S, Konrad M, Chubanov V, Gudermann T. TRPM6 and TRPM7–gatekeepers of human magnesium metabolism. Biochim Biophys Acta. 2007;1772(8):813–21. 10.1016/j.bbadis.2007.03.009. 17481860 10.1016/j.bbadis.2007.03.009

[71] Chamniansawat S, Suksridechacin N, Thongon N. Current opinion on the regulation of small intestinal magnesium absorption. World J Gastroenterol. 2023;29(2):332–42. 10.3748/wjg.v29.i2.332. 36687126 10.3748/wjg.v29.i2.332 PMC9846944

[72] Zofková I, Kancheva RL. The relationship between magnesium and calciotropic hormones. Magnes Res. 1995;8(1):77–84. 7669510

[73] Rondón LJ. Nutritional factors affecting magnesium bioavailability: a narrative review. Biol Trace Elem Res. 2026;204(2):1181–92. 10.1007/s12011-025-04739-2. 40668512 10.1007/s12011-025-04739-2

[74] Adomako EA, Yu ASL. Magnesium disorders: core curriculum 2024. Am J Kidney Dis. 2024;83(6):803–15. 10.1053/j.ajkd.2023.10.017. 38372687 10.1053/j.ajkd.2023.10.017

[75] de Rouffignac C, Morel F, Moss N, Roinel N. Micropuncture study of water and electrolyte movements along the loop of Henle in psammomys with special reference to magnesium, calcium and phosphorus. Pflugers Arch. 1973;344(4):309–26. 10.1007/bf00592784. 4798168 10.1007/BF00592784

[76] Ray E, Mohan K, Ahmad S, Wolf MTF. Physiology of a forgotten electrolyte-magnesium disorders. Adv Kidney Dis Health. 2023;30(2):148–63. 10.1053/j.akdh.2022.12.001. 36868730 10.1053/j.akdh.2022.12.001 PMC10291516

[77] Groenestege WM, Hoenderop JG, van den Heuvel L, Knoers N, Bindels RJ. The epithelial Mg2 + channel transient receptor potential melastatin 6 is regulated by dietary Mg2 + content and estrogens. J Am Soc Nephrol. 2006;17(4):1035–43. 10.1681/asn.2005070700. 16524949 10.1681/ASN.2005070700

[78] Manger B, Schett G. Magnesium disorders. N Engl J Med. 2024;391(7):668–9. 10.1056/NEJMc2408312. 39141871 10.1056/NEJMc2408312

[79] Qiao W, Wong KHM, Shen J, Wang W, Wu J, Li J, et al. TRPM7 kinase-mediated immunomodulation in macrophage plays a central role in magnesium ion-induced bone regeneration. Nat Commun. 2021;12(1):2885. 10.1038/s41467-021-23005-2. 34001887 10.1038/s41467-021-23005-2 PMC8128914

[80] Montezano AC, Zimmerman D, Yusuf H, Burger D, Chignalia AZ, Wadhera V, et al. Vascular smooth muscle cell differentiation to an osteogenic phenotype involves TRPM7 modulation by magnesium. Hypertension. 2010;56(3):453–62. 10.1161/hypertensionaha.110.152058. 20696983 10.1161/HYPERTENSIONAHA.110.152058

[81] Ab Rahim SN, Nordin N, Wan Omar WFA, Zulkarnain S, Kumar S, Sinha S, et al. The laboratory and clinical perspectives of magnesium imbalance. Cureus. 2023;15(12):e49835. 10.7759/cureus.49835. 38045630 10.7759/cureus.49835 PMC10693313

[82] Soori R, Dixit A, Tewari P. Refractory hypokalemia while weaning off bypass. Ann Card Anaesth. 2018;21(3):311–2. 10.4103/aca.ACA_196_17. 30052224 10.4103/aca.ACA_196_17 PMC6078024

[83] Rosanoff A, West C, Elin RJ, Micke O, Baniasadi S, Barbagallo M, et al. Recommendation on an updated standardization of serum magnesium reference ranges. Eur J Nutr. 2022;61(7):3697–706. 10.1007/s00394-022-02916-w. 35689124 10.1007/s00394-022-02916-w PMC9186275

[84] Safavi M, Honarmand A. Admission hypomagnesemia–impact on mortality or morbidity in critically ill patients. Middle East J Anaesthesiol. 2007;19(3):645–60. 18044292

[85] Rosanoff A, Weaver CM, Rude RK. Suboptimal magnesium status in the United States: are the health consequences underestimated? Nutr Rev. 2012;70(3):153–64. 10.1111/j.1753-4887.2011.00465.x. 22364157 10.1111/j.1753-4887.2011.00465.x

[86] Fan L, Zhu X, Rosanoff A, Costello RB, Yu C, Ness R, et al. Magnesium depletion score (MDS) predicts risk of systemic inflammation and cardiovascular mortality among US adults. J Nutr. 2021;151(8):2226–35. 10.1093/jn/nxab138. 34038556 10.1093/jn/nxab138 PMC8349125

[87] Zhang X, Xia J, Del Gobbo LC, Hruby A, Dai Q, Song Y. Serum magnesium concentrations and all-cause, cardiovascular, and cancer mortality among U.S. adults: results from the NHANES I epidemiologic follow-up study. Clin Nutr. 2018;37(5):1541–9. 10.1016/j.clnu.2017.08.021. 28890274 10.1016/j.clnu.2017.08.021

[88] Welch AA, Fransen H, Jenab M, Boutron-Ruault MC, Tumino R, Agnoli C, et al. Variation in intakes of calcium, phosphorus, magnesium, iron and potassium in 10 countries in the European Prospective Investigation into Cancer and Nutrition study. Eur J Clin Nutr. 2009;63(Suppl 4):S101-21. 10.1038/ejcn.2009.77. 19888269 10.1038/ejcn.2009.77

[89] Bain LK, Myint PK, Jennings A, Lentjes MA, Luben RN, Khaw KT, et al. The relationship between dietary magnesium intake, stroke and its major risk factors, blood pressure and cholesterol, in the EPIC-Norfolk cohort. Int J Cardiol. 2015;196:108–14. 10.1016/j.ijcard.2015.05.166. 26082204 10.1016/j.ijcard.2015.05.166 PMC6284795

[90] Lin B, Alexander R, Fritzen R, Mills S, Stewart AJ, McCowan C. Abnormal plasma/serum magnesium, copper, and zinc concentrations associate with the future development of cardiovascular diseases. Nutrients. 2025. 10.3390/nu17091447. 40362756 10.3390/nu17091447 PMC12073607

[91] Kieboom BCT, Ligthart S, Dehghan A, Kurstjens S, de Baaij JHF, Franco OH, et al. Serum magnesium and the risk of prediabetes: a population-based cohort study. Diabetologia. 2017;60(5):843–53. 10.1007/s00125-017-4224-4. 28224192 10.1007/s00125-017-4224-4 PMC6518103

[92] Spiga R, Mannino GC, Mancuso E, Averta C, Paone C, Rubino M, et al. Are circulating Mg(2+) levels associated with glucose tolerance profiles and incident type 2 diabetes? Nutrients. 2019. 10.3390/nu11102460. 31615167 10.3390/nu11102460 PMC6835462

[93] Lanehart D, Uzoma C, Zaman MS, Akimbekov NS, Lopez-Alvarenga JC, Razzaque MS. Maternal and fetal magnesium balance: impacts and implications. Adv Exp Med Biol. 2026;1493:133–9. 10.1007/978-3-032-04357-3_11. 41219604 10.1007/978-3-032-04357-3_11

[94] Dalton LM, Dm NF, Gaydadzhieva GT, Mazurkiewicz OM, Leeson H, Wright CP. Magnesium in pregnancy. Nutr Rev. 2016;74(9):549–57. 10.1093/nutrit/nuw018. 27445320 10.1093/nutrit/nuw018

[95] Anastasiou IA, Kounatidis D, Honka MJ, Vallianou NG, Rebelos E, Karamanolis NN, et al. Metabolomic alterations in patients with obesity and the impact of metabolic bariatric surgery: insights for future research. Metabolites. 2025. 10.3390/metabo15070434. 40710534 10.3390/metabo15070434 PMC12300167

[96] Varga P, Lehoczki A, Fekete M, Jarecsny T, Kryczyk-Poprawa A, Zábó V, et al. The role of magnesium in depression, migraine, Alzheimer’s disease, and cognitive health: a comprehensive review. Nutrients. 2025. 10.3390/nu17132216. 40647320 10.3390/nu17132216 PMC12252419

[97] Botturi A, Ciappolino V, Delvecchio G, Boscutti A, Viscardi B, Brambilla P. The role and the effect of magnesium in mental disorders: a systematic review. Nutrients. 2020. 10.3390/nu12061661. 32503201 10.3390/nu12061661 PMC7352515

[98] Ehrenpreis ED, Jarrouj G, Meader R, Wagner C, Ellis M. A comprehensive review of hypomagnesemia. Dis Mon. 2022;68(2):101285. 10.1016/j.disamonth.2021.101285. 34511254 10.1016/j.disamonth.2021.101285

[99] Arpaci D, Tocoglu AG, Ergenc H, Korkmaz S, Ucar A, Tamer A. Associations of serum magnesium levels with diabetes mellitus and diabetic complications. Hippokratia. 2015;19(2):153–7. 27418765 PMC4938107

[100] Srinutta T, Chewcharat A, Takkavatakarn K, Praditpornsilpa K, Eiam-Ong S, Jaber BL, et al. Proton pump inhibitors and hypomagnesemia: A meta-analysis of observational studies. Med (Baltim). 2019;98(44):e17788. 10.1097/md.0000000000017788. 10.1097/MD.0000000000017788 PMC6946416 31689852

[101] Nakamura T, Arai Y, Tando Y, Terada A, Yamada N, Tsujino M, et al. Effect of omeprazole on changes in gastric and upper small intestine pH levels in patients with chronic pancreatitis. Clin Ther. 1995;17(3):448–59. 10.1016/0149-2918(95)80110-3. 7585849 10.1016/0149-2918(95)80110-3

[102] Li M, Jiang J, Yue L. Functional characterization of homo- and heteromeric channel kinases TRPM6 and TRPM7. J Gen Physiol. 2006;127(5):525–37. 10.1085/jgp.200609502. 16636202 10.1085/jgp.200609502 PMC2151519

[103] Ohara K, Masuda T, Murakami T, Imai T, Yoshizawa H, Nakagawa S, et al. Effects of the sodium-glucose cotransporter 2 inhibitor dapagliflozin on fluid distribution: a comparison study with furosemide and tolvaptan. Nephrology (Carlton). 2019;24(9):904–11. 10.1111/nep.13552. 30578654 10.1111/nep.13552

[104] De Waele L, Van Gaal PJ, Abramowicz D. Electrolytes disturbances after kidney transplantation. Acta Clin Belg. 2019;74(1):48–52. 10.1080/17843286.2018.1549193. 30482110 10.1080/17843286.2018.1549193

[105] Jiang DM, Dennis K, Steinmetz A, Clemons M, Asmis TR, Goodwin RA, et al. Management of Epidermal Growth Factor Receptor Inhibitor-Induced Hypomagnesemia: A Systematic Review. Clin Colorectal Cancer. 2016;15(3):e117–23. 10.1016/j.clcc.2016.02.011. 26961757 10.1016/j.clcc.2016.02.011

[106] Oronsky B, Caroen S, Oronsky A, Dobalian VE, Oronsky N, Lybeck M, et al. Electrolyte disorders with platinum-based chemotherapy: mechanisms, manifestations and management. Cancer Chemother Pharmacol. 2017;80(5):895–907. 10.1007/s00280-017-3392-8. 28730291 10.1007/s00280-017-3392-8 PMC5676816

[107] McMahon KR, Harel-Sterling M, Pizzi M, Huynh L, Hessey E, Zappitelli M. Long-term renal follow-up of children treated with cisplatin, carboplatin, or ifosfamide: a pilot study. Pediatr Nephrol. 2018;33(12):2311–20. 10.1007/s00467-018-3976-5. 30218190 10.1007/s00467-018-3976-5

[108] Hu B, Huang S, Yin L. The cytokine storm and COVID-19. J Med Virol. 2021;93(1):250–6. 10.1002/jmv.26232. 32592501 10.1002/jmv.26232 PMC7361342

[109] Anghel L, Manole C, Nechita A, Tatu AL, Ștefănescu BI, Nechita L, et al. Calcium, phosphorus and magnesium abnormalities associated with COVID-19 infection, and beyond. Biomedicines. 2023. 10.3390/biomedicines11092362. 37760804 10.3390/biomedicines11092362 PMC10525362

[110] Nielsen FH. Magnesium deficiency and increased inflammation: current perspectives. J Inflamm Res. 2018;11:25–34. 10.2147/jir.S136742. 29403302 10.2147/JIR.S136742 PMC5783146

[111] Rodríguez-Morán M, Simental Mendía LE, Zambrano Galván G, Guerrero-Romero F. The role of magnesium in type 2 diabetes: a brief based-clinical review. Magnes Res. 2011;24(4):156–62. 10.1684/mrh.2011.0299. 22198525 10.1684/mrh.2011.0299

[112] Yin Y, Cheng Y, Zullo AR, Shao Y, Sheriff HM, Faselis C, et al. Serum magnesium, prescribed magnesium replacement and cardiovascular events in adults with type 2 diabetes: a national cohort study in U.S. veterans. Nutrients. 2025. 10.3390/nu17132067. 40647173 10.3390/nu17132067 PMC12251466

[113] Pitliya A, Vasudevan SS, Batra V, Patel MB, Desai A, Nethagani S, et al. Global prevalence of hypomagnesemia in type 2 diabetes mellitus - a comprehensive systematic review and meta-analysis of observational studies. Endocrine. 2024;84(3):842–51. 10.1007/s12020-023-03670-7. 38159172 10.1007/s12020-023-03670-7

[114] Pelczyńska M, Moszak M, Bogdański P. The role of magnesium in the pathogenesis of metabolic disorders. Nutrients. 2022. 10.3390/nu14091714. 35565682 10.3390/nu14091714 PMC9103223

[115] Castellanos-Gutiérrez A, Sánchez-Pimienta TG, Carriquiry A, da Costa THM, Ariza AC. Higher dietary magnesium intake is associated with lower body mass index, waist circumference and serum glucose in Mexican adults. Nutr J. 2018;17(1):114. 10.1186/s12937-018-0422-2. 30518394 10.1186/s12937-018-0422-2 PMC6282375

[116] de Sousa Melo SR, Dos Santos LR, da Cunha Soares T, Cardoso BEP, da Silva Dias TM, Morais JBS, et al. Participation of magnesium in the secretion and signaling pathways of insulin: an updated review. Biol Trace Elem Res. 2022;200(8):3545–53. 10.1007/s12011-021-02966-x. 35666386 10.1007/s12011-021-02966-x

[117] Chua FB, Cinco JE, Paz-Pacheco E. Efficacy of magnesium supplementation on glycemic control in type 2 diabetes patients: a meta-analysis. J ASEAN Fed Endocr Soc. 2017;32(1):38–45. 10.15605/jafes.032.01.07. 33442083 10.15605/jafes.032.01.07 PMC7784187

[118] Han H, Fang X, Wei X, Liu Y, Jin Z, Chen Q, et al. Dose-response relationship between dietary magnesium intake, serum magnesium concentration and risk of hypertension: a systematic review and meta-analysis of prospective cohort studies. Nutr J. 2017;16(1):26. 10.1186/s12937-017-0247-4. 28476161 10.1186/s12937-017-0247-4 PMC5420140

[119] Rotter I, Kosik-Bogacka D, Dołęgowska B, Safranow K, Karakiewicz B, Laszczyńska M. Relationship between serum magnesium concentration and metabolic and hormonal disorders in middle-aged and older men. Magnes Res. 2015;28(3):99–107. 10.1684/mrh.2015.0391. 26507751 10.1684/mrh.2015.0391

[120] Argeros Z, Xu X, Bhandari B, Harris K, Touyz RM, Schutte AE. Magnesium supplementation and blood pressure: a systematic review and meta-analysis of randomized controlled trials. Hypertension. 2025;82(11):1844–56. 10.1161/hypertensionaha.125.25129. 41000008 10.1161/HYPERTENSIONAHA.125.25129 PMC12529988

[121] Costello RB, Elin RJ, Rosanoff A, Wallace TC, Guerrero-Romero F, Hruby A, et al. Perspective: the case for an evidence-based reference interval for serum magnesium: the time has come. Adv Nutr. 2016;7(6):977–93. 10.3945/an.116.012765. 28140318 10.3945/an.116.012765 PMC5105038

[122] Rosique-Esteban N, Guasch-Ferré M, Hernández-Alonso P, Salas-Salvadó J. Dietary magnesium and cardiovascular disease: a review with emphasis in epidemiological studies. Nutrients. 2018. 10.3390/nu10020168. 29389872 10.3390/nu10020168 PMC5852744

[123] Nakata K, Toida T, Kurita N, Abe M, Hanafusa N, Joki N. Hypomagnesemia in peritoneal and hybrid dialysis: prevalence, predictors, and association with atrial fibrillation. Clin Exp Nephrol. 2025. 10.1007/s10157-025-02810-9. 41452545 10.1007/s10157-025-02810-9

[124] Huang CY, Yang CC, Hung KC, Jiang MY, Huang YT, Hwang JC, et al. Association between hypomagnesemia and mortality among dialysis patients: a systematic review and meta-analysis. PeerJ. 2022;10:e14203. 10.7717/peerj.14203. 36248710 10.7717/peerj.14203 PMC9563282

[125] Liu H, Wang R. Associations between the serum magnesium and all-cause or cardiovascular mortality in chronic kidney disease and end-stage renal disease patients: a meta-analysis. Medicine (Baltimore). 2021;100(45):e27486. 10.1097/md.0000000000027486. 34766558 10.1097/MD.0000000000027486 PMC8589258

[126] DiNicolantonio JJ, O’Keefe JH, Wilson W. Subclinical magnesium deficiency: a principal driver of cardiovascular disease and a public health crisis. Open Heart. 2018;5(1):e000668. 10.1136/openhrt-2017-000668. 29387426 10.1136/openhrt-2017-000668 PMC5786912

[127] Shahi A, Aslani S, Ataollahi M, Mahmoudi M. The role of magnesium in different inflammatory diseases. Inflammopharmacology. 2019;27(4):649–61. 10.1007/s10787-019-00603-7. 31172335 10.1007/s10787-019-00603-7

[128] Barker-Haliski M, White HS. Glutamatergic mechanisms associated with seizures and epilepsy. Cold Spring Harb Perspect Med. 2015;5(8):a022863. 10.1101/cshperspect.a022863. 26101204 10.1101/cshperspect.a022863 PMC4526718

[129] Kirkland AE, Sarlo GL, Holton KF. The role of magnesium in neurological disorders. Nutrients. 2018. 10.3390/nu10060730. 29882776 10.3390/nu10060730 PMC6024559

[130] Tarleton EK, Littenberg B. Magnesium intake and depression in adults. J Am Board Fam Med. 2015;28(2):249–56. 10.3122/jabfm.2015.02.140176. 25748766 10.3122/jabfm.2015.02.140176

[131] Cheungpasitporn W, Thongprayoon C, Mao MA, Srivali N, Ungprasert P, Varothai N, et al. Hypomagnesaemia linked to depression: a systematic review and meta-analysis. Intern Med J. 2015;45(4):436–40. 10.1111/imj.12682. 25827510 10.1111/imj.12682

[132] Chen F, Wang J, Cheng Y, Li R, Wang Y, Chen Y, et al. Magnesium and cognitive health in adults: A Systematic review and meta-analysis. Adv Nutr. 2024;15(8):100272. 10.1016/j.advnut.2024.100272. 39009081 10.1016/j.advnut.2024.100272 PMC11362647

[133] Du K, Zheng X, Ma ZT, Lv JY, Jiang WJ, Liu MY. Association of circulating magnesium levels in patients with Alzheimer’s disease from 1991 to 2021: a systematic review and meta-analysis. Front Aging Neurosci. 2021;13:799824. 10.3389/fnagi.2021.799824. 35082658 10.3389/fnagi.2021.799824 PMC8784804

[134] Hajhashemy Z, Shirani F, Askari G. Dietary magnesium intake in relation to depression in adults: a GRADE-assessed systematic review and dose-response meta-analysis of epidemiologic studies. Nutr Rev. 2025;83(2):217–29. 10.1093/nutrit/nuae056. 38812090 10.1093/nutrit/nuae056

[135] Boyle NB, Lawton C, Dye L. The effects of magnesium supplementation on subjective anxiety and stress-a systematic review. Nutrients. 2017. 10.3390/nu9050429. 28445426 10.3390/nu9050429 PMC5452159

[136] Klein GL. The role of calcium in inflammation-associated bone resorption. Biomolecules. 2018. 10.3390/biom8030069. 30071694 10.3390/biom8030069 PMC6163591

[137] Chang J, Yu D, Ji J, Wang N, Yu S, Yu B. The association between the concentration of serum magnesium and postmenopausal osteoporosis. Front Med (Lausanne). 2020;7:381. 10.3389/fmed.2020.00381. 32850896 10.3389/fmed.2020.00381 PMC7417435

[138] Groenendijk I, van Delft M, Versloot P, van Loon LJC, de Groot L. Impact of magnesium on bone health in older adults: a systematic review and meta-analysis. Bone. 2022;154:116233. 10.1016/j.bone.2021.116233. 34666201 10.1016/j.bone.2021.116233

[139] Bonilla M, Workeneh BT, Uppal NN. Hypomagnesemia in patients with cancer: the forgotten ion. Semin Nephrol. 2022;42(6):151347. 10.1016/j.semnephrol.2023.151347. 37086496 10.1016/j.semnephrol.2023.151347

[140] Polter EJ, Onyeaghala G, Lutsey PL, Folsom AR, Joshu CE, Platz EA, et al. Prospective association of serum and dietary magnesium with colorectal cancer incidence. Cancer Epidemiol Biomarkers Prev. 2019;28(8):1292–9. 10.1158/1055-9965.Epi-18-1300. 31167754 10.1158/1055-9965.EPI-18-1300 PMC6677594

[141] Bagheri A, Naghshi S, Sadeghi O, Larijani B, Esmaillzadeh A. Total, dietary, and supplemental magnesium intakes and risk of all-cause, cardiovascular, and cancer mortality: a systematic review and dose-response meta-analysis of prospective cohort studies. Adv Nutr. 2021;12(4):1196–210. 10.1093/advances/nmab001. 33684200 10.1093/advances/nmab001 PMC8321838

[142] Shah SC, Dai Q, Zhu X, Peek RM Jr., Smalley W, Roumie C, et al. <article-title update="added">Associations between calcium and magnesium intake and the risk of incident gastric cancer: a prospective cohort analysis of the National Institutes of Health‐American Association of Retired Persons (NIH‐AARP) diet and health study. Int J Cancer. 2020;146(11):2999–3010. 10.1002/ijc.32659. 10.1002/ijc.32659 PMC7048631 31472027

[143] Shah SC, Zhu X, Dai Q, Peek RM, Shrubsole MJ. Magnesium intake is associated with a reduced risk of incident liver cancer, based on an analysis of the NIH-American Association of Retired Persons (NIH-AARP) diet and health study prospective cohort. Am J Clin Nutr. 2021;113(3):630–8. 10.1093/ajcn/nqaa326. 33330925 10.1093/ajcn/nqaa326 PMC8324220

[144] Lin T, Bi C, Song Y, Guo H, Liu L, Zhou Z, et al. Plasma Magnesium Concentrations and Risk of Incident Cancer in Adults with Hypertension: A Nested Case-Control Study. Ann Nutr Metab. 2020;76(5):304–12. 10.1159/000510214. 33271534 10.1159/000510214

[145] Blaszczyk U, Duda-Chodak A. Magnesium: its role in nutrition and carcinogenesis. Rocz Panstw Zakl Hig. 2013;64(3):165–71. 24325082

[146] Saglietti F, Girombelli A, Marelli S, Vetrone F, Balzanelli MG, Tabaee Damavandi P. Role of magnesium in the intensive care unit and immunomodulation: a literature review. Vaccines (Basel). 2023. 10.3390/vaccines11061122. 37376511 10.3390/vaccines11061122 PMC10304084

[147] van Kempen T, Deixler E. SARS-CoV-2: influence of phosphate and magnesium, moderated by vitamin D, on energy (ATP) metabolism and on severity of COVID-19. Am J Physiol Endocrinol Metab. 2021;320(1):E2-e6. 10.1152/ajpendo.00474.2020. 33174766 10.1152/ajpendo.00474.2020 PMC7816430

[148] Tian J, Tang L, Liu X, Li Y, Chen J, Huang W, et al. Populations in low-magnesium areas were associated with higher risk of infection in COVID-19’s early transmission: a nationwide retrospective cohort study in the United States. Nutrients. 2022. 10.3390/nu14040909. 35215558 10.3390/nu14040909 PMC8875017

[149] La Carrubba A, Veronese N, Di Bella G, Cusumano C, Di Prazza A, Ciriminna S, et al. Prognostic value of magnesium in COVID-19: findings from the COMEPA study. Nutrients. 2023. 10.3390/nu15040830. 36839188 10.3390/nu15040830 PMC9966815

[150] Segev A, Sagir A, Matetzky S, Segev A, Atar S, Shechter M. Admission serum magnesium levels is associated with short and long-term clinical outcomes in COVID-19 patients. Nutrients. 2023. 10.3390/nu15092016. 37432174 10.3390/nu15092016 PMC10180728

[151] Guerrero-Romero F, Mercado M, Rodriguez-Moran M, Ramírez-Renteria C, Martínez-Aguilar G, Marrero-Rodríguez D, et al. Magnesium-to-calcium ratio and mortality from COVID-19. Nutrients. 2022. 10.3390/nu14091686. 35565654 10.3390/nu14091686 PMC9101802

[152] Solanki J, Runwal K, Beke N, Bahulikar A, Phalgune D. Serum magnesium levels in critically ill patients on admission in ICU and its correlation with outcome. J Assoc Physicians India. 2022;70(5):11–2. 35598126

[153] Van Laecke S. Hypomagnesemia and hypermagnesemia. Acta Clin Belg. 2019;74(1):41–7. 10.1080/17843286.2018.1516173. 30220246 10.1080/17843286.2018.1516173

[154] Hansen BA, Bruserud Ø. Hypomagnesemia in critically ill patients. J Intensive Care. 2018;6:21. 10.1186/s40560-018-0291-y. 29610664 10.1186/s40560-018-0291-y PMC5872533

[155] Aal-Hamad AH, Al-Alawi AM, Kashoub MS, Falhammar H. Hypermagnesemia in clinical practice. Medicina (Kaunas). 2023. 10.3390/medicina59071190. 37512002 10.3390/medicina59071190 PMC10384947

[156] Trapani V, Rosanoff A, Baniasadi S, Barbagallo M, Castiglioni S, Guerrero-Romero F, et al. The relevance of magnesium homeostasis in COVID-19. Eur J Nutr. 2022;61(2):625–36. 10.1007/s00394-021-02704-y. 34687321 10.1007/s00394-021-02704-y PMC8540865

[157] Uwitonze AM, Razzaque MS. Role of magnesium in vitamin D activation and function. J Am Osteopath Assoc. 2018;118(3):181–9. 10.7556/jaoa.2018.037. 29480918 10.7556/jaoa.2018.037

[158] Barbagallo M, Veronese N, Dominguez LJ. Magnesium in aging, health and diseases. Nutrients. 2021. 10.3390/nu13020463. 33573164 10.3390/nu13020463 PMC7912123

[159] Reddy ST, Soman SS, Yee J. Magnesium balance and measurement. Adv Chronic Kidney Dis. 2018;25(3):224–9. 10.1053/j.ackd.2018.03.002. 29793660 10.1053/j.ackd.2018.03.002

[160] Ayuk J, Gittoes NJ. Contemporary view of the clinical relevance of magnesium homeostasis. Ann Clin Biochem. 2014;51(Pt 2):179–88. 10.1177/0004563213517628. 24402002 10.1177/0004563213517628

[161] Elin RJ. Assessment of magnesium status for diagnosis and therapy. Magnes Res. 2010;23(4):S194–8. 10.1684/mrh.2010.0213. 20736141 10.1684/mrh.2010.0213

[162] Arnaud MJ. Update on the assessment of magnesium status. Br J Nutr. 2008;99(Suppl 3):S24–36. 10.1017/s000711450800682x. 18598586 10.1017/S000711450800682X

[163] Pollock N, Chakraverty R, Taylor I, Killer SC. An 8-year analysis of magnesium status in elite international track & field athletes. J Am Coll Nutr. 2020;39(5):443–9. 10.1080/07315724.2019.1691953. 31829845 10.1080/07315724.2019.1691953

[164] Stendig-Lindberg G, Shapiro Y, Epstein Y, Galun E, Schonberger E, Graff E, et al. Changes in serum magnesium concentration after strenuous exercise. J Am Coll Nutr. 1987;6(1):35–40. 10.1080/07315724.1987.10720163. 3453693 10.1080/07315724.1987.10720163

[165] Saad AS, Ismail NS, Gaber NS, Elzanfaly ES. A chemically modified solid-state sensor for magnesium(II) ions and esomeprazole magnesium potentiometric assay. RSC Adv. 2023;13(3):1995–2003. 10.1039/d2ra06839g. 36712625 10.1039/d2ra06839g PMC9832439

[166] Bazzano G, Galazzi A, Giusti GD, Panigada M, Laquintana D. The order of draw during blood collection: a systematic literature review. Int J Environ Res Public Health. 2021. 10.3390/ijerph18041568. 33562241 10.3390/ijerph18041568 PMC7915193

[167] Sanmartí J, Robles-Guirado JA, Jose-Cunilleras E, Bassols A. Sample stability and heparin interference in ionized calcium and ionized magnesium measurements in horses using the Stat Profile Prime Plus co-oximetry electrolyte analyzer. Vet Clin Pathol. 2023;52(2):252–60. 10.1111/vcp.13200. 36746672 10.1111/vcp.13200

[168] Dent A, Selvaratnam R. Measuring magnesium - Physiological, clinical and analytical perspectives. Clin Biochem. 2022;105–106:1–15. 10.1016/j.clinbiochem.2022.04.001. 35381264 10.1016/j.clinbiochem.2022.04.001

[169] Ryan MF, Barbour H. Magnesium measurement in routine clinical practice. Ann Clin Biochem. 1998;35(Pt 4):449–59. 10.1177/000456329803500401. 9681049 10.1177/000456329803500401

[170] Lutz NW, Bernard M. Multiparametric quantification of heterogeneity of metal ion concentrations, as demonstrated for [Mg(2+)] by way of (31)P MRS. J Magn Reson. 2018;294:71–82. 10.1016/j.jmr.2018.06.016. 30015125 10.1016/j.jmr.2018.06.016

[171] Ansu Baidoo VY, Cara KC, Dickinson SL, Brown AW, Wallace TC, Chung M, et al. Systematic review and meta-analysis to estimate a reference range for circulating ionized magnesium concentrations in adult populations. J Nutr. 2023;153(12):3458–71. 10.1016/j.tjnut.2023.10.006. 37844840 10.1016/j.tjnut.2023.10.006

[172] Sutton RA, Domrongkitchaiporn S. Abnormal renal magnesium handling. Miner Electrolyte Metab. 1993;19(4–5):232–40. 8264509

[173] Workinger JL, Doyle RP, Bortz J. Challenges in the diagnosis of magnesium status. Nutrients. 2018. 10.3390/nu10091202. 30200431 10.3390/nu10091202 PMC6163803

[174] Costello RB, Nielsen F. Interpreting magnesium status to enhance clinical care: key indicators. Curr Opin Clin Nutr Metab Care. 2017;20(6):504–11. 10.1097/mco.0000000000000410. 28806179 10.1097/MCO.0000000000000410 PMC5812344

[175] Ayuk J, Gittoes NJ. Treatment of hypomagnesemia. Am J Kidney Dis. 2014;63(4):691–5. 10.1053/j.ajkd.2013.07.025. 24100128 10.1053/j.ajkd.2013.07.025

[176] Liamis G, Hoorn EJ, Florentin M, Milionis H. An overview of diagnosis and management of drug-induced hypomagnesemia. Pharmacol Res Perspect. 2021;9(4):e00829. 10.1002/prp2.829. 34278747 10.1002/prp2.829 PMC8287009

[177] Kisters K. What is the correct magnesium supplement? Magnes Res. 2013;26(1):41–2. 10.1684/mrh.2012.0326. 23708889 10.1684/mrh.2012.0326

[178] Dai LJ, Raymond L, Friedman PA, Quamme GA. Mechanisms of amiloride stimulation of Mg2 + uptake in immortalized mouse distal convoluted tubule cells. Am J Physiol. 1997;272(2 Pt 2):F249–56. 10.1152/ajprenal.1997.272.2.F249. 9124403 10.1152/ajprenal.1997.272.2.F249

[179] Hicks MA, Tyagi A, Magnesium Sulfate. 2023. In: StatPearls. Treasure Island (FL): StatPearls Publishing; 2025; PMID: 32119440. 32119440

[180] Asbaghi O, Moradi S, Kashkooli S, Zobeiri M, Nezamoleslami S, Hojjati Kermani MA, et al. The effects of oral magnesium supplementation on glycaemic control in patients with type 2 diabetes: a systematic review and dose-response meta-analysis of controlled clinical trials. Br J Nutr. 2022;128(12):2363–72. 10.1017/s0007114521005201. 35045911 10.1017/S0007114521005201

[181] Veronese N, Pizzol D, Smith L, Dominguez LJ, Barbagallo M. Effect of magnesium supplementation on inflammatory parameters: a meta-analysis of randomized controlled trials. Nutrients. 2022. 10.3390/nu14030679. 35277037 10.3390/nu14030679 PMC8838086

[182] Veronese N, Demurtas J, Pesolillo G, Celotto S, Barnini T, Calusi G, et al. Magnesium and health outcomes: an umbrella review of systematic reviews and meta-analyses of observational and intervention studies. Eur J Nutr. 2020;59(1):263–72. 10.1007/s00394-019-01905-w. 30684032 10.1007/s00394-019-01905-w

[183] Sadeghpour M, Bejani A, Kupaei MH, Majd SJA, Najafi A, Fakhari S, et al. Unraveling the mechanisms of magnesium supplementation in alleviating chronic kidney disease complications and progression: balancing risks and benefits. Biol Trace Elem Res. 2025;203(5):2539–49. 10.1007/s12011-024-04368-1. 39256329 10.1007/s12011-024-04368-1

[184] Vermeulen EA, Vervloet MG. Magnesium administration in chronic kidney disease. Nutrients. 2023. 10.3390/nu15030547. 36771254 10.3390/nu15030547 PMC9920010

[185] Moabedi M, Aliakbari M, Erfanian S, Milajerdi A. Magnesium supplementation beneficially affects depression in adults with depressive disorder: a systematic review and meta-analysis of randomized clinical trials. Front Psychiatry. 2023;14:1333261. 10.3389/fpsyt.2023.1333261. 38213402 10.3389/fpsyt.2023.1333261 PMC10783196

[186] Matek Sarić M, Sorić T, Juko Kasap Ž, Lisica Šikić N, Mavar M, Andruškienė J, et al. Magnesium: health effects, deficiency burden, and future public health directions. Nutrients. 2025. 10.3390/nu17223626. 41305676 10.3390/nu17223626 PMC12655508

[187] Kröse JL, de Baaij JHF. Magnesium biology. Nephrol Dial Transplant. 2024;39(12):1965–75. 10.1093/ndt/gfae134. 38871680 10.1093/ndt/gfae134 PMC11648962

[188] Liu M, Dudley SC Jr. Beyond ion homeostasis: hypomagnesemia, transient receptor potential melastatin channel 7, mitochondrial function, and inflammation. Nutrients. 2023. 10.3390/nu15183920. 10.3390/nu15183920 PMC10536927 37764704

[189] Colaneri-Day S, Rosanoff A. Clinical guideline for detection and management of magnesium deficiency in ambulatory care. Nutrients. 2025. 10.3390/nu17050887. 40077757 10.3390/nu17050887 PMC11901669

